# Plant-based production of a protective vaccine antigen against the bovine parasitic nematode *Ostertagia ostertagi*

**DOI:** 10.1038/s41598-023-47480-3

**Published:** 2023-11-22

**Authors:** Laurens Zwanenburg, Jimmy Borloo, Bregt Decorte, Myrna J. M. Bunte, Sanaz Mokhtari, Sonia Serna, Niels-C. Reichardt, Leen J. M. Seys, Angela van Diepen, Arjen Schots, Ruud H. P. Wilbers, Cornelis H. Hokke, Edwin Claerebout, Peter Geldhof

**Affiliations:** 1 Laboratory of Parasitology, Department of Translational Physiology, Infectiology and Public Health, Faculty of Veterinary Medicine, Ghent University, Salisburylaan 133, B-9820 Merelbeke, Belgium; 2 Laboratory of Nematology, Department of Plant Sciences, Wageningen University & Research, Droevendaalsesteeg 1, 6708 PB Wageningen, The Netherlands; 3 Department of Parasitology, Leiden University Medical Center, Albinusdreef 2, 2333 ZA Leiden, The Netherlands; 4 Glycotechnology Laboratory, Center for Cooperative Research in Biomaterials (CIC biomaGUNE), Basque Research and Technology Alliance (BRTA), Paseo de Miramon 194, 20014 Donostia San Sebastián, Spain; 5 CIBER-BBN, Paseo Miramón 194, 20014 San Sebastian, Spain

**Keywords:** Protein vaccines, Parasitic infection

## Abstract

The development of effective recombinant vaccines against parasitic nematodes has been challenging and so far mostly unsuccessful. This has also been the case for *Ostertagia ostertagi*, an economically important abomasal nematode in cattle, applying recombinant versions of the protective native activation-associated secreted proteins (ASP). To gain insight in key elements required to trigger a protective immune response, the protein structure and N-glycosylation of the native ASP and a non-protective *Pichia pastoris* recombinant ASP were compared. Both antigens had a highly comparable protein structure, but different N-glycan composition. After mimicking the native ASP N-glycosylation via the expression in *Nicotiana benthamiana* plants, immunisation of calves with these plant-produced recombinants resulted in a significant reduction of 39% in parasite egg output, comparable to the protective efficacy of the native antigen. This study provides a valuable workflow for the development of recombinant vaccines against other parasitic nematodes.

## Introduction

Gastrointestinal nematode infections pose a major health concern for both humans and animals. Over one billion people are affected by gastrointestinal nematodes, mostly children living in developing countries^1,2^. In the veterinary field, basically every animal is either infected or at risk of being infected, compromising animal health, welfare and, in the case of farm animals, productivity^3,4^. Controlling these nematode infections primarily entails periodic mass anthelmintic drug administration. In the long term, a control strategy solely reliant on anthelmintics is not viable due to the growing global trend of anthelmintic resistance^5,6^. With limited new drug compounds in the pipeline, there is strong interest for alternative and sustainable control measures. Immunological control of nematode infections through vaccination is considered promising with regards to sustainability and cost-effectiveness^7,8^. Unfortunately, progress in vaccine development has been limited, with only two commercial vaccines available on the market for animals. These are vaccines against the bovine lungworm *Dictyocaulus viviparus* (Bovilis Huskvac™) and against the sheep gastrointestinal nematode *Haemonchus contortus* (Barbervax™/Wirevax™), utilizing irradiated larvae and crude antigen mixtures, respectively. This approach is considered impractical for many nematode species due to the difficulty of obtaining sufficient quantities of larvae, worms or purified worm material^9,10^. For this reason, a large number of recombinant produced subunit vaccines have been evaluated against a range of gastrointestinal nematodes. Unfortunately, very few of these vaccines induced a sufficient level of protection to consider further commercial development, often attributed to inappropriate recombinant expression^10^.

The development of an experimental vaccine against the bovine abomasal nematode *Ostertagia ostertagi* has faced similar problems. This vaccine is based on activation-associated secreted protein, Oo-ASP-1, purified from the excretory-secretory (ES) material of adult *O. ostertagi* worms^11^. Immunisation of calves with this antigen resulted in a significant reduction in faecal egg output ranging from 56–74% in several studies^11–13^. In contrast, recombinant versions of the native Oo-ASP-1 have been unsuccessful in eliciting a protective immune response. Previous studies have demonstrated that recombinants produced in *Escherichia coli* and insect cells induce minimal cross-reactive antibody responses to the native antigens^14^. More recently, a recombinant Oo-ASP-1 produced in *Pichia pastoris* was able to trigger a cross-reactive antibody response but failed to provide protection against a controlled *O. ostertagi* challenge infection. Notably, the capacity of the *P. pastoris*-expressed Oo-ASP-1 to trigger a local humoral and cellular response was significantly lower than that of the native antigen^13^.

The overall objective of the current study was to identify the structural features of the native Oo-ASP-1 that are necessary for the induction of a protective immune response and use the information to steer recombinant expression. The first aim was to analyse and compare protein folding and N-glycosylation of the native and recombinant Oo-ASP-1 versions and evaluate the impact of potential differences on antibody recognition. The second aim was to incorporate the missing key elements via an appropriate expression system and produce new recombinant versions of Oo-ASP-1. These were subsequently evaluated for their immunostimulatory and protective capacity as a vaccine antigen.

## Results

### Allelic variation of native Oo-ASP-1 does not influence secondary protein structure and has no implication on antibody recognition

**Figure 1:**
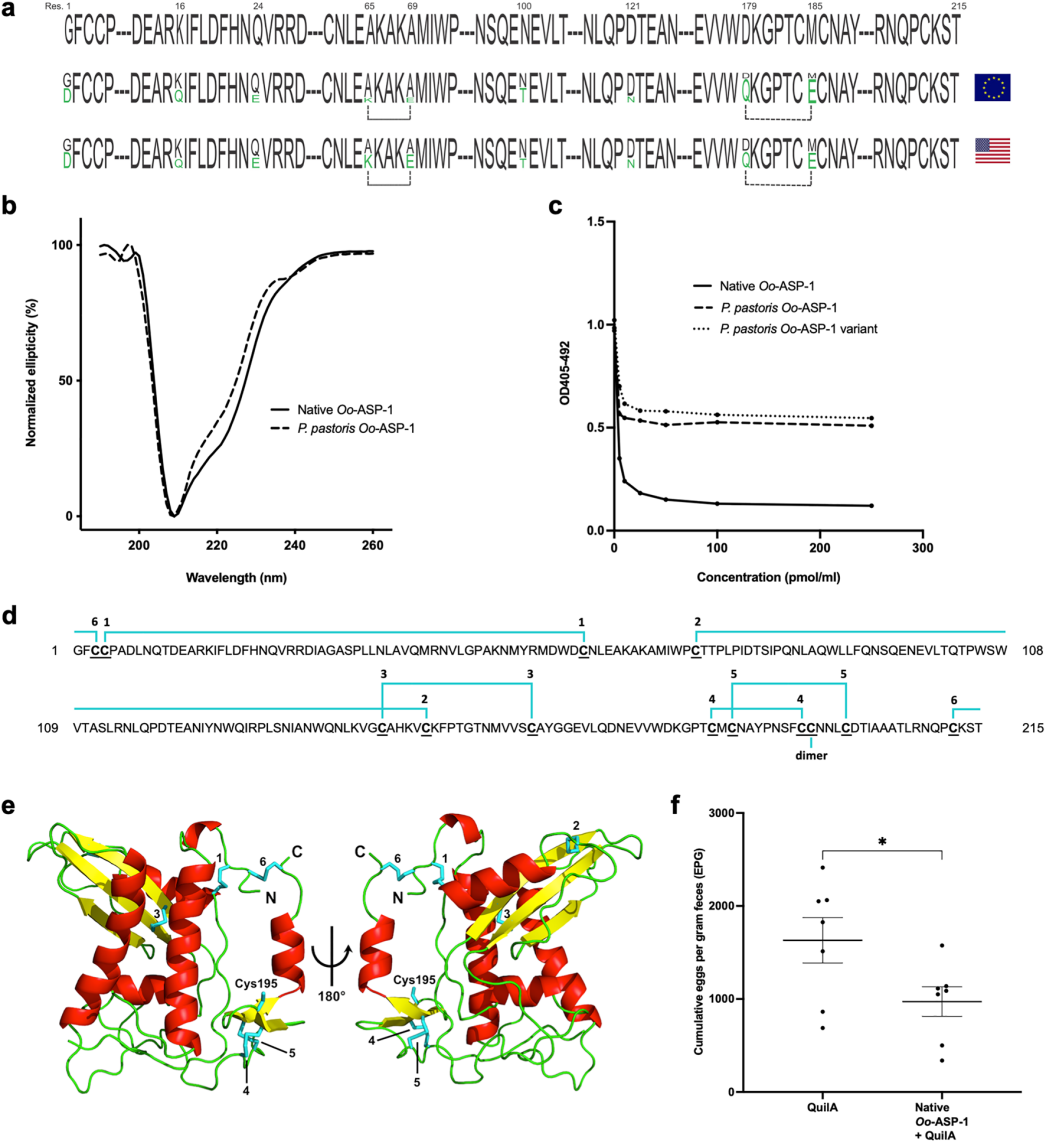
The impact of protein structure on antibody recognition and vaccine efficacy. (**a**) The Oo-ASP-1 amino acid sequence of the *Pichia pastoris*-expressed recombinant version (top), and native protein from European (middle) and North-American (bottom) isolates is partially presented. A total of nine positions in the ASP sequence were found to show sequence diversity. The corresponding residues are shown in black/green with the size of the letter reflecting its relative presence. Amino acid residue numbers prone to sequence diversity are indicated at the top. Dotted lines indicate residues with *quasi* identical polymorphism percentages. (**b**) Circular dichroism spectra of native Oo-ASP-1 (intact line) and *P. pastoris* recombinant (dashed line), normalized to allow comparison. (**c**) Competition ELISA showing binding preference of antibodies from calves immunised with native Oo-ASP-1 towards native Oo-ASP-1 (intact line), *P. pastoris* recombinant (dashed line) or a variant of the *P. pastoris* recombinant with included polymorphisms (dotted line). (**d**) Amino acid sequence of recombinant Oo-ASP-1 with the six intramolecular disulphide bonds numbered (1–6), and the cysteine involved in dimer formation (dimer). (**e**) *Pichia pastoris* recombinant Oo-ASP-1 structure^17^ showing the intramolecular disulphide bonds corresponding to panel *A*. (**f**) Immunisation-challenge study in cattle displaying *O. ostertagi* faecal egg output in calves immunised with size-exclusion purified native Oo-ASP-1 + QuilA (n = 7) versus QuilA-adjuvant controls (n = 7), presented as total cumulative eggs per gram faeces (EPG). Data are presented as mean values + / − standard error of the mean. *P* values were calculated using a one-tailed Mann–Whitney test. *P < 0.05, **P < 0.01 and ***P < 0.001 versus QuilA-adjuvant control. The experiments presented in (**a**), (**b**), (**c**) and (**f**) were conducted once. Data in (**d**) is based on two experiments. Source data is available upon request.

The native antigen is purified from the excretory/secretory (ES) material secreted by thousands of *O. ostertagi* worms, with the result that allelic variants of Oo-ASP-1 are potentially present in the native antigen preparation. Such variation is not present in the *P. pastoris* produced ASP as it is based on a single Oo-ASP-1 coding sequence. To investigate this, Oo-ASP-1 RNA was extracted from a pool of North-American and European *O. ostertagi* worms, converted into cDNA and bidirectionally sequenced. The analysis revealed nine amino acid residues highly susceptible to polymorphisms, which were all conserved in both isolates (Fig. 1a).

As this allelic variation may influence secondary structural features, circular dichroism (CD) experiments were subsequently conducted on both native and *P. pastoris* recombinant Oo-ASP-1. The CD spectra for both molecules were almost identical with a major peak at 208 nm, and an additional shoulder at 221 nm (Fig. 1b), indicating a high α-helical content in both samples, indicative of a comparable secondary protein structure. High α-helical content is a characteristic feature of the superfamily of cysteine-rich secretory proteins, antigen 5, and pathogenesis-related 1 proteins (CAP) to which the ASPs belong^15–17^. To further evaluate the impact of allelic variation on antibody recognition, two recombinant versions that carried all amino-acid substitutions were produced in *P. pastoris* and tested in a competition ELISA. In this assay, serum from calves immunised with native Oo-ASP-1 was pre-incubated with different concentrations of either native Oo-ASP-1 or one of the *P. pastoris* recombinants. The formed antibody-antigen complexes were transferred onto native Oo-ASP-1 coated ELISA plates allowing the antibodies to either maintain their binding to the pre-incubation antigen or dissociate and bind to the antigen coated on the plate. Antibody binding to the coated antigen was measured and expressed in optical density (OD) values. As displayed in Fig. 1c, native Oo-ASP-1 was capable of *quasi* completely disrupting the initially formed complexes between antibodies and both *P. pastoris* recombinant versions, demonstrated by the higher OD values compared to native Oo-ASP-1. This is an indicator that the introduction of the allelic variants in the recombinants had no significant impact on antibody recognition.

### Comparable protein folding and oligomerisation between native and *P. pastoris* Oo-ASP-1

ASPs are known to bear multiple signature disulphide bonds^18,19^, dictating the characteristic folding of these proteins. The cysteine-containing tryptic peptides on the native Oo-ASP-1 sequence that were anticipated to be involved in disulphide bonding are depicted in Fig. 1d. A liquid chromatography-mass spectrometry (LC–MS) approach was employed to elucidate the disulphide bonding patterns of native and *P. pastoris* Oo-ASP-1. The native antigen was found to have intramolecular disulphide bonds 3, 4 and 5, but no thiol-carrying peptides corresponding to bonds 1, 2 and 6 were detected (Supplementary Table 1). The presence of all CAP protein-hallmark intramolecular disulphide bonds was confirmed for the *P. pastoris* recombinant (Supplementary Table 1), consistent with previous observations from the 3D-structure (Fig. 1e)^17^. It was postulated that the inconsistency for native Oo-ASP-1 compared to the prediction data may be largely due to the purification method of this antigen, as it involved the reduction and reoxidation of disulphide bonds induced by dithiothreitol (DTT), followed by thiol-Sepharose affinity and ion exchange chromatography.

Therefore, native Oo-ASP-1 was purified from *O. ostertagi* ES material via size-exclusion chromatography, omitting the reduction and oxidation steps, and subsequently included in the MS analyses. With this approach, all predicted intramolecular disulphide bonds were identified for the native antigen (Supplementary Table 1), indicating no differences in protein folding between the native Oo-ASP-1 and the *P. pastoris* recombinant. In terms of oligomerisation, it was previously reported that native Oo-ASP-1 forms a disulphide bridge-based dimer through oxidation of both monomer’s Cys195 residues^17^. Reducing and non-reducing gel analyses (Supplementary Figure 1) combined with mass spectrometric data confirmed that both native Oo-ASP-1 and the *P. pastoris* recombinant dimerize and that no aberrations in quaternary structure were further noted.

Previous immunisation studies in cattle were conducted with the thiol-Sepharose purified native Oo-ASP-1, consistently demonstrating the capacity of this antigen to provide protection against *O. ostertagi* infections^11–13^. Given the difference in disulphide bonding pattern, it was imperative to investigate whether the size-exclusion purified native Oo-ASP-1 could still induce protection. To evaluate this, a bovine immunisation-infection study (n = 7) was conducted comparing a group immunised with the size-exclusion purified native Oo-ASP-1 to a QuilA, a saponin-based adjuvant, control group. Over the course of the study, faecal egg output for the group immunised with Oo-ASP-1 was found to be lower, resulting in a 40% reduction in cumulative faecal egg counts (*P* < 0.05) (Fig. 1f). No differences in total worm burden were observed between the *Oo*-ASP-1 immunised and the QuilA control groups, but the Oo-ASP-1 immunised group showed a significantly higher percentage of inhibited L4-stage worms (*P* < 0.01) (Supplementary Table 2). These results are similar to the outcome obtained with the thiol-Sepharose purified Oo-ASP-1 and indicate that the disulphide bond patterning has no discernible impact on the antigen’s capacity to provide protection.

### Differences in N-glycosylation between native and *P. pastoris* Oo-ASP-1 impact glycan-directed antibody recognition.

Following the confirmation of the similar protein structure of the *P. pastoris* recombinant and native Oo-ASP-1, the subsequent study sought to analyse the N-glycosylation of both antigens and assess the impact of potential disparities on antibody recognition. Previous studies showed that native Oo-ASP-1 bears two FucGalGlcNAc_3_Man_3_ moieties per monomer at asparagine residues 9 and 37 (Asn9 and Asn37)^20^. In contrast, the *P. pastoris* recombinants were expressed in a GnM5 strain^21^, which adds predominantly GalGlcNAc_3_Man_5_ moieties to the aforementioned Asn residues^17^.

To evaluate the relevance of N-glycans in antibody recognition, both versions of Oo-ASP-1 were subjected to enzymatic deglycosylation using Peptide:N-glycosidase F (PNGase F), with the aim to compare them in a competition ELISA. In agreement with previous observations^20^, Oo-ASP-1 could not be fully deglycosylated without denaturing and thus structurally disrupting the protein, hampering further competition ELISA analyses.

**Figure 2:**
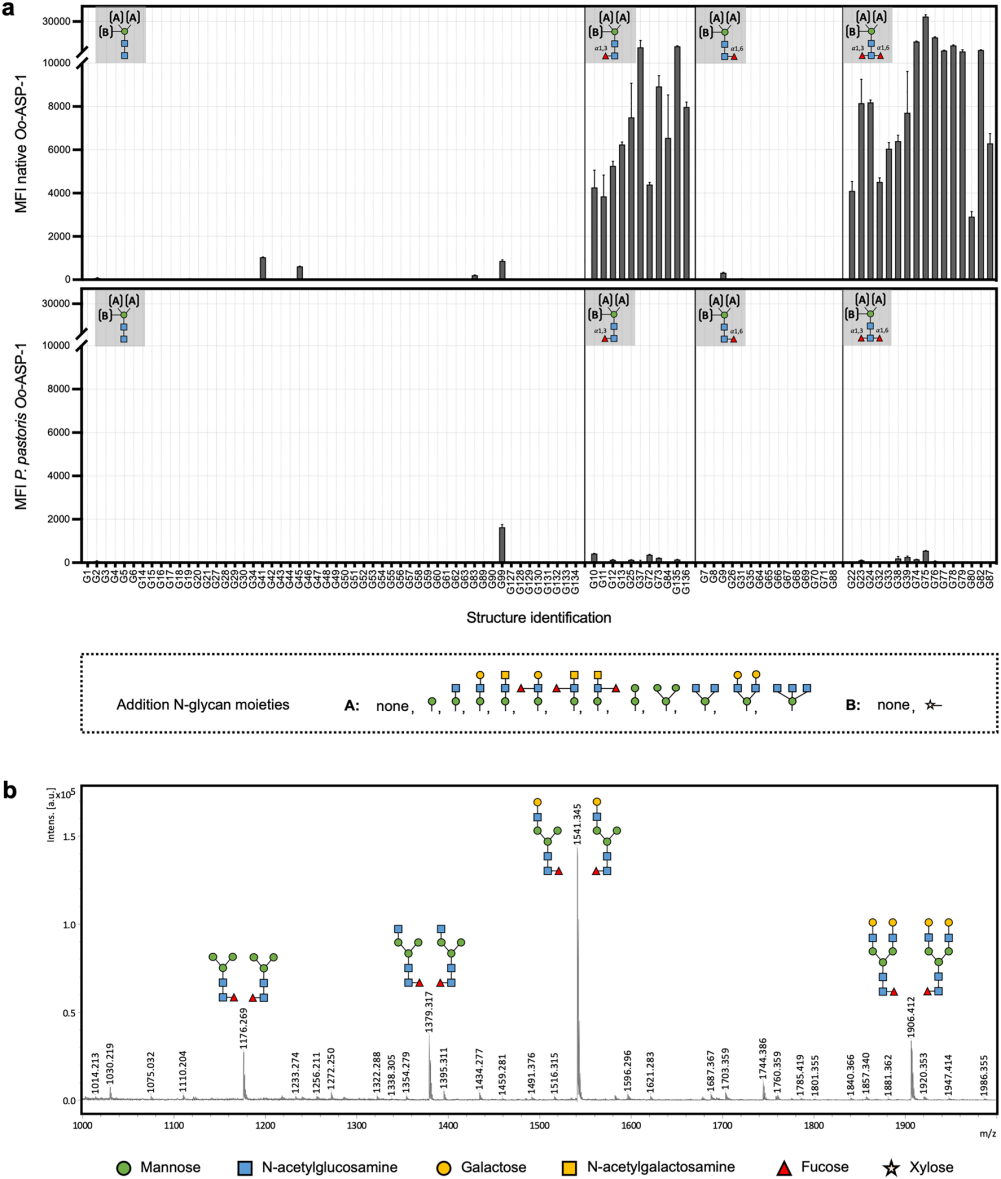
Glycan microarrays and mass-spectrometric N-glycan evaluation of native Oo-ASP-1. (**a**) Glycan microarrays displaying glycan recognition by pooled (n = 11) native antigen-specific antibodies from calves immunised with native Oo-ASP-1 (upper panel) or *Pichia pastoris* Oo-ASP-1 (lower panel), expressed as Mean Fluorescence Index (MFI). The X-axis contains the structure identification codes for the glycans, as previously published^22^. Glycan structures are organised based on core fucosylation type, depicted in the top-left corner of each frame. Further modifications are indicated by “*A”* and “*B”*, and displayed in the legend below. (**b**) Mass-spectrometry on PNGase A released N-glycans from native Oo-ASP-1. Differentiation between core α1,3-fucose and core α1,6-fucose was not made in this figure, hence the double displayed N-glycan structures with both core fucose linkage-types. Monoisotopic masses of measured signals are indicated, and the proposed glycan structures are depicted based on the Consortium for Functional Glycomics (CFG) nomenclature. The X-axis displays the mass to charge ratio (m/z), whilst the Y-axis displays the relative intensity in arbitrary units (intens. [a.u.]). Data in (**a**) are presented as mean values + / − standard error of the mean of four technical replicates. Experiments presented in (**a**) and (**b**) were conducted once. Source data is available upon request.

As an alternative to evaluate the role of Oo-ASP-1 associated N-glycans in antibody recognition, microarrays carrying a variety of synthetic N-glycans, presented in a previous study^22^, were screened with pooled native Oo-ASP-1-specific antibodies, immunopurified from serum of calves immunised with either native Oo-ASP-1, *P. pastoris* Oo-ASP-1 or QuilA. It was demonstrated that ASP-specific antibodies were able to bind to several N-glycans in the absence of the ASP-protein, albeit only from native Oo-ASP-1 immunised calves (Fig. 2a). Antibodies from native Oo-ASP-1 immunised calves distinctly recognised N-glycans that carried core α1,3-fucose, while recognition of N-glycans that lacked core fucose or carried only core α1,6-fucose was limited. In contrast, ASP-specific antibodies from *P. pastoris* recombinant-immunised calves exhibited minimal recognition of N-glycans on the array. QuilA-immunised calves lacked ASP-specific antibodies and thus microarray assay signals were negative (Supplementary Figure 2).

While the array data suggests the presence of core α1,3-fucose on N-glycans of native Oo-ASP-1, this was previously not observed^20^. This could be explained by the fact that the N-glycan release in that study was conducted with PNGase F, which is unable to remove N-glycans that contain an α1,3-fucosylated core^23^. To reassess the N-glycosylation of native Oo-ASP-1, the denatured protein was treated with peptide:N-glycosidase A (PNGase A) to release all N-glycans prior to MS analysis. Released N-glycans were paucimannosidic, lacking further branching, or complex-type, where the trimannosyl core element was substituted with a GlcNAc or GalGlcNAc moiety (Fig. 2b), in each case also containing a fucose residue in the overall composition, in line with previous findings^20^. Hydrofluoric acid (HF) treatment followed by MS subsequently confirmed the presence of both α1,3-fucose and α1,6-fucose, but never on the same glycans (Supplementary Figure 3).

### Newly expressed *Nicotiana benthamiana* Oo-ASP-1 competes with native Oo-ASP-1 for antibody binding

**Figure 3:**
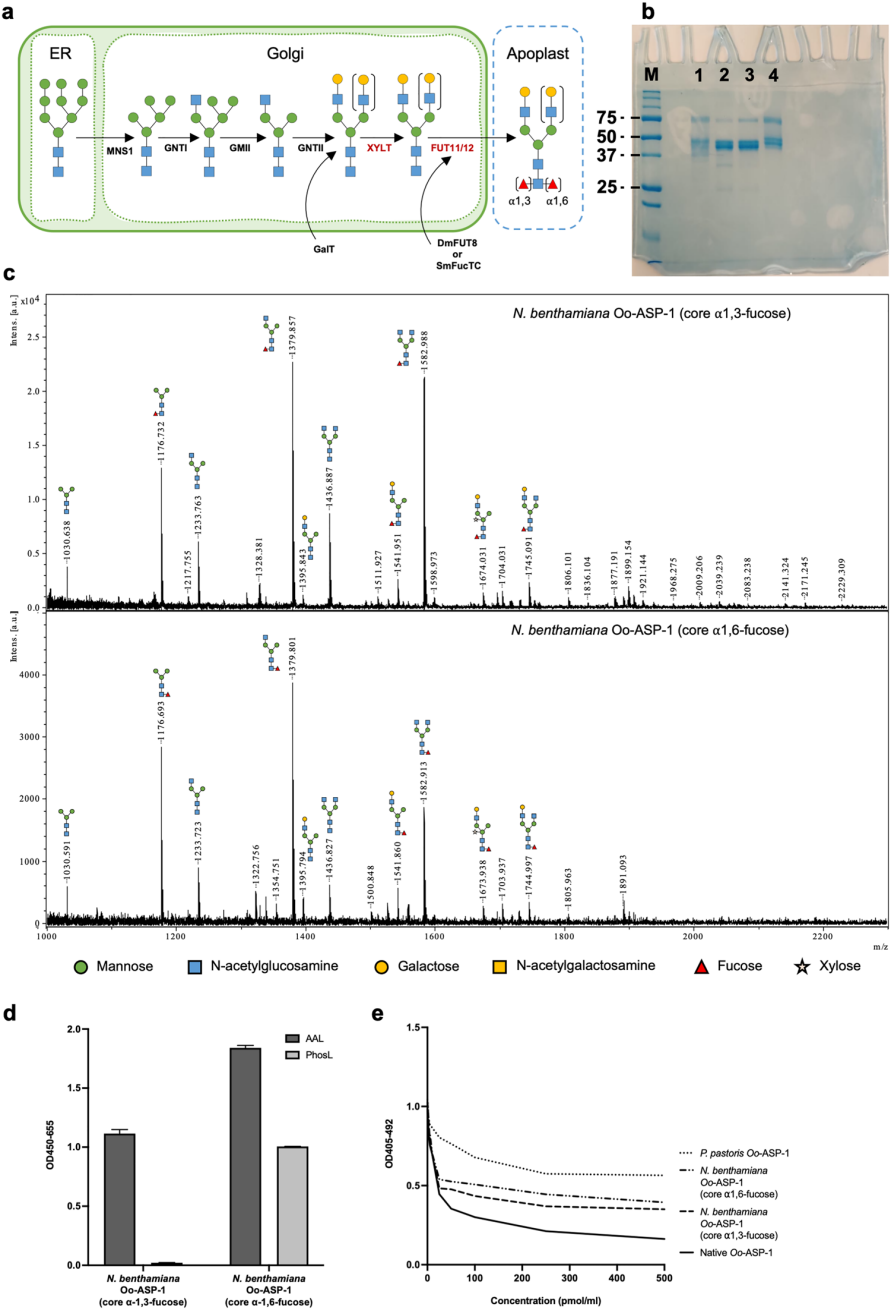
Production of *Nicotiana benthamiana* recombinant Oo-ASP-1. (**a**) Schematic overview of the N-glycosylation pathway in different compartments of *N. benthamiana*, modified by the expression of *Danio rerio* β1,4-galactosyltransferase (GalT), *Drosophila melanogaster* fucosyltransferase 8 (DmFUT8) or *Schistosoma mansoni* fucosyltransferase C (SmFucTC), resulting in hybrid-type N-glycosylation with either core α1,3-fucose or core α1,6-fucose. The suppressed Xylosyltransferase (XYLT) and Fucosyltransferase 11/12 (FUT11/12) are displayed in red, while mannosidase 1 (MNS1), N-acetylglucosaminyltransferase I (GNTI), Golgi mannosidase II (GMII) and N-acetylglucosaminyltransferase II (GNTII) remained active. (**b**) SDS-PAGE under non-reducing conditions showing the migration patterns for Precision Plus Protein™ standard (M), native Oo-ASP-1 (lane 1), *N. benthamiana* Oo-ASP-1 with core α1,3-fucose (lane 2), *N. benthamiana* Oo-ASP-1 with core α1,6-fucose (lane 3) and *P. pastoris* Oo-ASP-1 (lane 4). Molecular weight of 25, 37, 50 and 75 kDa protein bands are displayed on the left side of the figure. The uncropped version of the gel is available in Supplementary Figure 8. (**c**) Mass-spectrometry (MS) analysis of the N-glycosylation profile of *N. benthamiana* ASP with core α1,3-fucose (upper panel) and core α1,6-fucose (lower panel). The X-axis displays the mass to charge ratio (m/z), whilst the Y-axis displays the relative intensity in arbitrary units (intens. [a.u.]). (**d**) Lectin assay to determine core fucosylation-type on both *N. benthamiana* recombinants, facilitated via core α1,3/α1,6 fucose-binding *Aleuria aurantia* Lectin (AAL) and core α1,6-fucose-specific *Pholiota squarrosa* lectin (PhosL). Data are presented as mean values + / − standard error of the mean. (**e**) A pooled serum sample from calves immunised with native Oo-ASP-1 was pre-incubated with either native Oo-ASP-1, *N. benthamiana* Oo-ASP-1 with core α1,3-fucose, *N. benthamiana* Oo-ASP-1 with core α1,6-fucose or *Pichia pastoris* Oo-ASP-1, and evaluated in a competition ELISA. Data are presented as mean values based on two technical replicates per data point. Data from (**b**), (**c**), (**d**) and (**e**) are representative of two independent experiments conducted on two separately produced batches of *N. benthamiana* recombinants. Source data is available upon request.

Given that native Oo-ASP-1 is characterised by N-glycans carrying either core α1,3-fucose or core α1,6-fucose, two recombinant versions, each containing complex-type N-glycans with galactosylated branches and one of the fucose linkages, were synthesised in β1,2-xylosyltransferase and α1,3-fucosyltransferase down-regulated (ΔXT/FT) *N. benthamiana*. The plant’s leaves were infiltrated with *Agrobacterium tumefaciens* containing transfer DNA for the Oo-ASP-1 antigen and specific glycan-processing enzymes (Fig. 3a). The expressed antigens were secreted in the plant’s apoplast fluid and harvested six days after infiltration via vacuum infiltration of an extraction buffer, followed by centrifugation.

To compare basic protein structure of the newly generated recombinants, SDS-PAGE analysis was conducted, which revealed that both recombinant glycoforms exhibited a migration pattern of approximately 50 kDa (Fig. 3b), consistent with native Oo-ASP-1. MS analysis to evaluate the N-glycosylation showed that the *N. benthamiana* recombinants carried a variation of N-glycans, including the mono-antennary N-glycans present on the native antigen (Fig. 3c). Importantly, core fucose was present on almost all N-glycans. Although the enzymes used for introducing core α1,3-fucose and core α1,6-fucose are highly specific, further confirmation of the correct linkage type was conducted via lectin-assays (Fig. 3d). Terminal galactosylation was low for both recombinants but was increased after affinity purification with agarose bound *Ricinus Communis* Agglutinin I (Supplementary Figure 4) leading to an N-glycosylation profile that more closely resembles that of native Oo-ASP-1. Post-purification, the most abundant N-glycans found on the *N. benthamiana* Oo-ASP-1 include FucGlcNAc_3_Man_3_, FucGalGlcNAc_3_Man_3_, FucGlcNAc_4_Man_3_, FucGalGlcNAc_4_Man_3_, FucGal_2_GlcNAc_4_Man_3_.

To evaluate the impact of N-glycosylation on ASP immunogenicity, it was attempted to produce *N. benthamiana* recombinants that completely lacked N-glycans. However, mutation of the N-glycosylation sites Asn9 and Asn37 resulted in complete elimination of ASP expression, most likely attributed to the function of N-glycosylation in protein folding and subsequent secretion in the apoplast fluid. Also, attempts to enzymatically remove N-glycans from the intact protein using PNGase F treatment led to incomplete deglycosylation, which was also observed for *P. pastoris* and previously for intact native Oo-ASP-1^20^.

The presence of shared antibody binding sites between the *N. benthamiana* recombinants and the native antigen was confirmed via indirect ELISA (Supplementary Figure 5), demonstrating a clear recognition of both *N. benthamiana* recombinants by serum from native Oo-ASP-1 immunised calves. Via a competition ELISA it was revealed that both glycoforms, especially the core α1,3-fucose variant, exhibited a stronger inhibition of antibody binding to native Oo-ASP-1 compared to the *P. pastoris* recombinant (Fig. 3e). These findings suggest a greater degree of antibody epitope overlap between native Oo-ASP-1 and the two *N. benthamiana* recombinants, with a particular contribution of the core α1,3-fucose epitope in line with the glycan array data (Fig. 2a).

### Immunisation with *N. benthamiana* Oo-ASP-1 results in a reduction in faecal egg output

With the newly obtained *N. benthamiana* recombinants, which better mimic the native Oo-ASP-1, a bovine immunisation and challenge study was conducted to evaluate their ability to protect calves against a controlled *O. ostertagi* infection. The recombinant vaccine comprised a 1:1 ratio of *N. benthamiana* recombinants (n = 8) with core α1,3-fucose and core α1,6-fucose, as both fucose types are also present on the native antigen, and its protective efficacy was assessed in comparison to native Oo-ASP-1 (n = 7) and a QuilA-adjuvant control group (n = 7).

**Figure 4:**
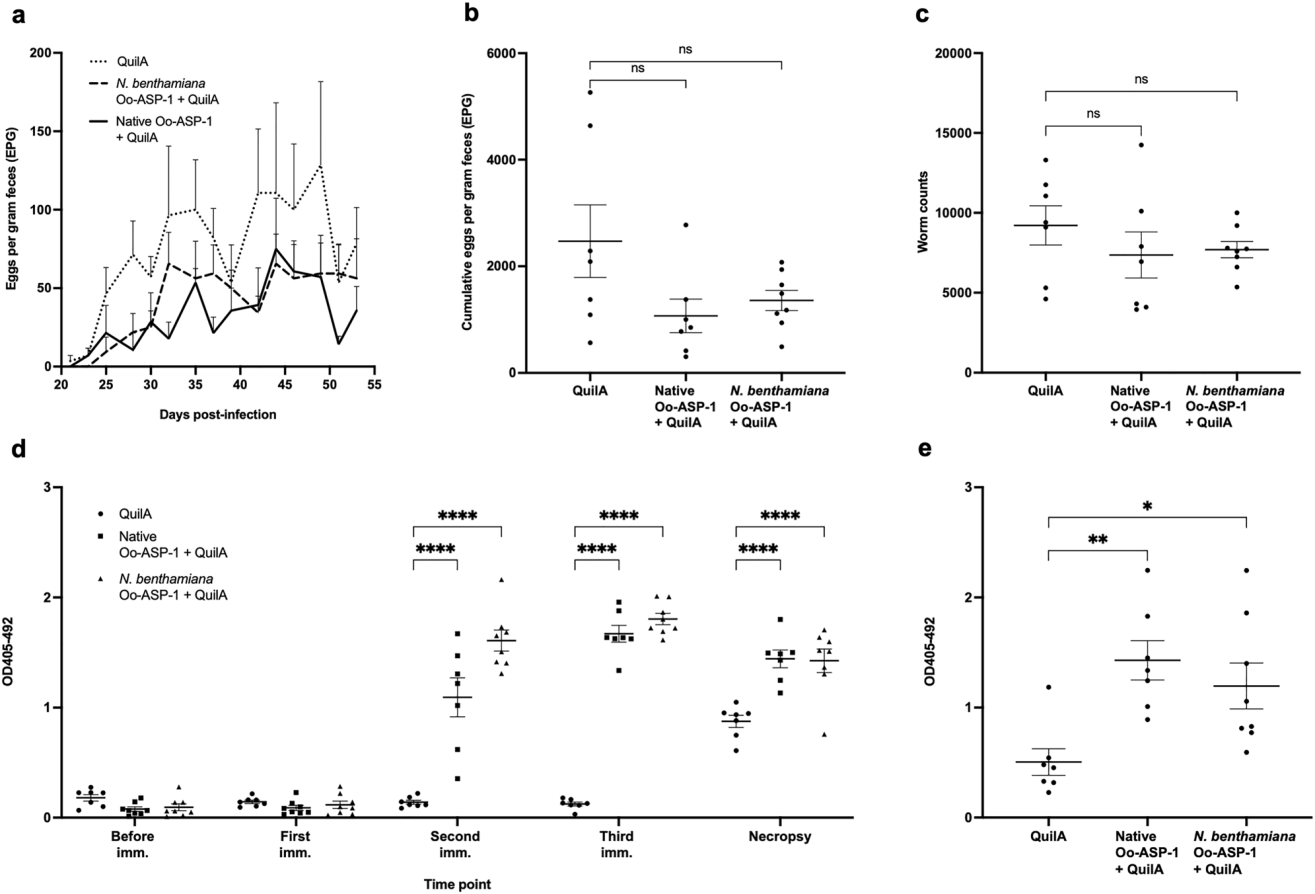
Immunisation-challenge study in calves with native Oo-ASP-1 and *Nicotiana benthamiana* Oo-ASP-1. Calves were immunised three times with either native Oo-ASP-1 + QuilA (n = 7), *Nicotiana benthamiana* Oo-ASP-1 + QuilA (n = 8) or QuilA alone (n = 7) prior to a trickle infection with L3-stage *Ostertagi ostertagi*. Evaluated parasitological parameters include (**a**) faecal egg output measured over time, (**b**) cumulative faecal egg output and (**c**) *O. ostertagi* worm counts at the time of necropsy. (**d**) The systemic IgG1 response to native Oo-ASP-1 in an ELISA was measured prior to immunisation (imm), one week after each immunisation and at necropsy, displayed in optical density 405 nm with background correction at 492 nm. (**e**) The mucosal IgG1 response to native Oo-ASP-1 was measured at time of necropsy, displayed in optical density 405 nm with background correction at 492 nm. Data are presented as mean values + / − standard error of the mean. *P* values for (**b**), (**c**) and (**e**) were calculated using a Kruskal–Wallis test with Dunn’s test for multiple comparison. *P* values for (**d**) were calculated using a Two-way ANOVA with Dunnet’s test for multiple comparison. Degrees of freedom for the numerator: 8. Degrees of freedom for the denominator: 97. F distribution value: 40.92. *P < 0.05, **P < 0.01, ***P < 0.001 and ****P < 0.0001 versus QuilA-adjuvant control. The experiments for all figures were conducted once. Source data is available upon request.

After three intramuscular immunisations followed by a trickle infection, the *N. benthamiana* group exhibited a decline in faecal egg output over time in a similar trend to that of the native Oo-ASP-1 group (Fig. 4a). This resulted in a reduction of the cumulative faecal egg output by 57% for the native Oo-ASP-1 group and 45% for the *N. benthamiana* recombinant group, compared to adjuvant-controls (Fig. 4b). Despite not achieving statistical significance (*P* > 0.05), likely due to the high variation in the adjuvant-control group, it was the first recombinant ASP that elicited a comparable reduction in faecal egg output to that of native Oo-ASP-1^13,14^. Immunisation with native Oo-ASP-1 or the *N. benthamiana* recombinant did not clearly impact (*P* > 0.05) the total worm burden (Fig. 4c) or length of male worms. Female worm length was significantly reduced in the native vaccine group (Table 1). The percentage of inhibited L4-stage worms was increased (*P* < 0.05) in the native Oo-ASP-1 group (Table 1).

**Table 1:** Overview of parasitological parameters obtained after the *Nicotiana benthamiana* Oo-ASP-1 immunisation-challenge study. n, number of animals; EPG, mean cumulative eggs per gram faeces; % L4, percentage of L4 worms observed in post-necropsy worm counting; Male and female worm length in µm.

Group	n	Cumulative EPG	Worm count	% L4	Male worm length (µm)	Female worm length (µm)
QuilA control	7	2471 (563 – 5263)	9214 (5300 – 13,300)	16 (2 – 32)	7519 (6990 – 7773)	9119 (8889 – 9378)
Size-exclusion purified native Oo-ASP-1	7	1070 (300 – 2775)	7364 (3950 – 14,250)	42 (14 – 83) *	7009 (6111 – 7477)	8622 (8449 – 8969) **
*N. benthamiana* Oo-ASP-1	8	1358 (488 – 2075)	7694 (5350 – 10,000)	26 (16 – 34)	7646 (7359 – 7972	9038 (8827 – 9228)

*P < 0.05; **P < 0.01.

All values represent arithmetic means (+ experimentally observed range).

After the second immunisation, a native Oo-ASP-1-reactive systemic IgG1 (Fig. 4d) and IgG2 (Supplementary Figure 6) response was induced by the *N. benthamiana* recombinant, comparable to the native Oo-ASP-1 group. Also, the recombinant vaccine was able to elicit a significant (*P* < 0.05) increase in native Oo-ASP-1-reactive mucosal IgG1 (Fig. 4e) and IgG2 (Supplementary Figure 6) compared to the control group, which had only been observed for calves immunised with native Oo-ASP-1 in previous studies. No differences (*P* > 0.05) in local IgA were noted between the three groups (Supplementary Figure 6).

**Figure 5:**
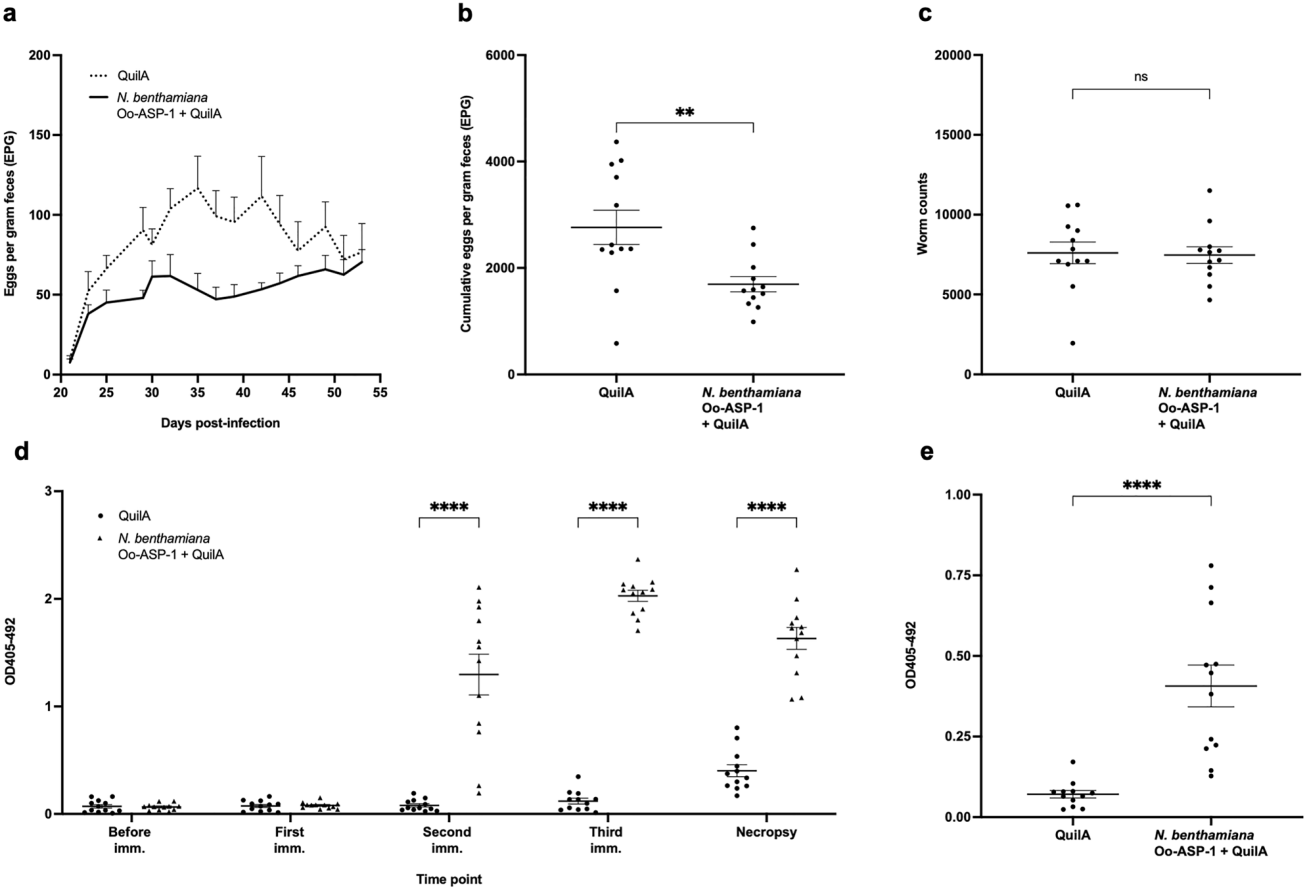
Immunisation-challenge study in calves with *Nicotiana benthamiana* Oo-ASP-1. Calves were immunised three times with either *Nicotiana benthamiana* Oo-ASP-1 + QuilA (n = 12) or QuilA alone (n = 12) prior to a trickle infection with L3-stage *Ostertagia ostertagi*. Evaluated parasitological parameters include (**a**) faecal egg output measured over time, (**b**) cumulative faecal egg output and (**c**) *O. ostertagi* worm counts at the time of necropsy. (**d**) The systemic IgG1 response to native Oo-ASP-1 in an ELISA was measured prior to immunisation (imm), one week after each immunisation and at necropsy, displayed in optical density 405 nm with background correction at 492 nm. (**e**) The mucosal IgG1 response to native Oo-ASP-1 was measured at time of necropsy, displayed in optical density 405 nm with background correction at 492 nm. Data are presented as mean values + / − standard error of the mean. *P* values for (**b**), (**c**) and (**e**) were calculated using a one-tailed Mann–Whitney test. *P* values for (**d**) were calculated using a Two-way ANOVA with Dunnet’s test for multiple comparison. Degrees of freedom for the numerator: 4. Degrees of freedom for the denominator: 110. F distribution value: 66.04. *P < 0.05, **P < 0.01, ***P < 0.001 and ****P < 0.0001 versus QuilA-adjuvant control. The experiments for all figures were conducted once. Source data is available upon request.

To increase the statistical power of these data, the immunisation-challenge study was repeated with larger group sizes (n = 12). In this study, the *N. benthamiana* recombinant with core α1,3/α1,6-fucose in a 1:1 ratio was compared to a QuilA-alone adjuvant group. Consistent with the initial study, immunisation with the *N. benthamiana* recombinant resulted in a decrease in faecal egg output over time (Fig. 5a) and a significant reduction of 39% (*P* < 0.01) in cumulative faecal egg excretion (Fig. 5b), validating the findings of the first study. No differences were observed between the two vaccine groups in the percentage of L4-stage worms (Table 2), total worm burden (Fig. 5c) or male and female worm length (Table 2).

**Table 2:** Overview of parasitological parameters obtained after the *Nicotiana benthamiana* Oo-ASP-1 immunisation-challenge study. n, number of animals; EPG, mean cumulative eggs per gram faeces; % L4, percentage of L4 worms observed in post-necropsy worm counting; Male and female worm length in µm.

Group	n	Cumulative EPG	Worm count	% L4	Male worm length (µm)	Female worm length (µm)
QuilA control	12	2762 (580 – 4368)	7608 (1950 – 10,600)	12 (3 – 49)	7483 (7143 – 7780)	9008 (8185 – 9553)
*N. benthamiana* Oo-ASP-1	12	1694 (985 – 2750) **	7467 (4650 – 11,500)	14 (2 – 30)	7390 (7059 – 7642)	8983 (8613 – 9238)

All values represent arithmetic means (+ experimentally observed range).

*P < 0.05; **P < 0.01.

The humoral immune response to the *N. benthamiana* recombinant was in line with the previous smaller group size study, with a clear native Oo-ASP-1-reactive systemic IgG1 (Fig. 5d) and IgG2 (Supplementary Figure 7) response after the second and third immunisations, respectively. The capacity of the *N. benthamiana* recombinant to induce a native Oo-ASP-1-reactive mucosal IgG1 (Fig. 5e) and IgG2 (Supplementary Figure 7) response was also confirmed, whereas local IgA did not differ between the *N. benthamiana* recombinant and QuilA groups (Supplementary Figure 7).

**Figure 6:**
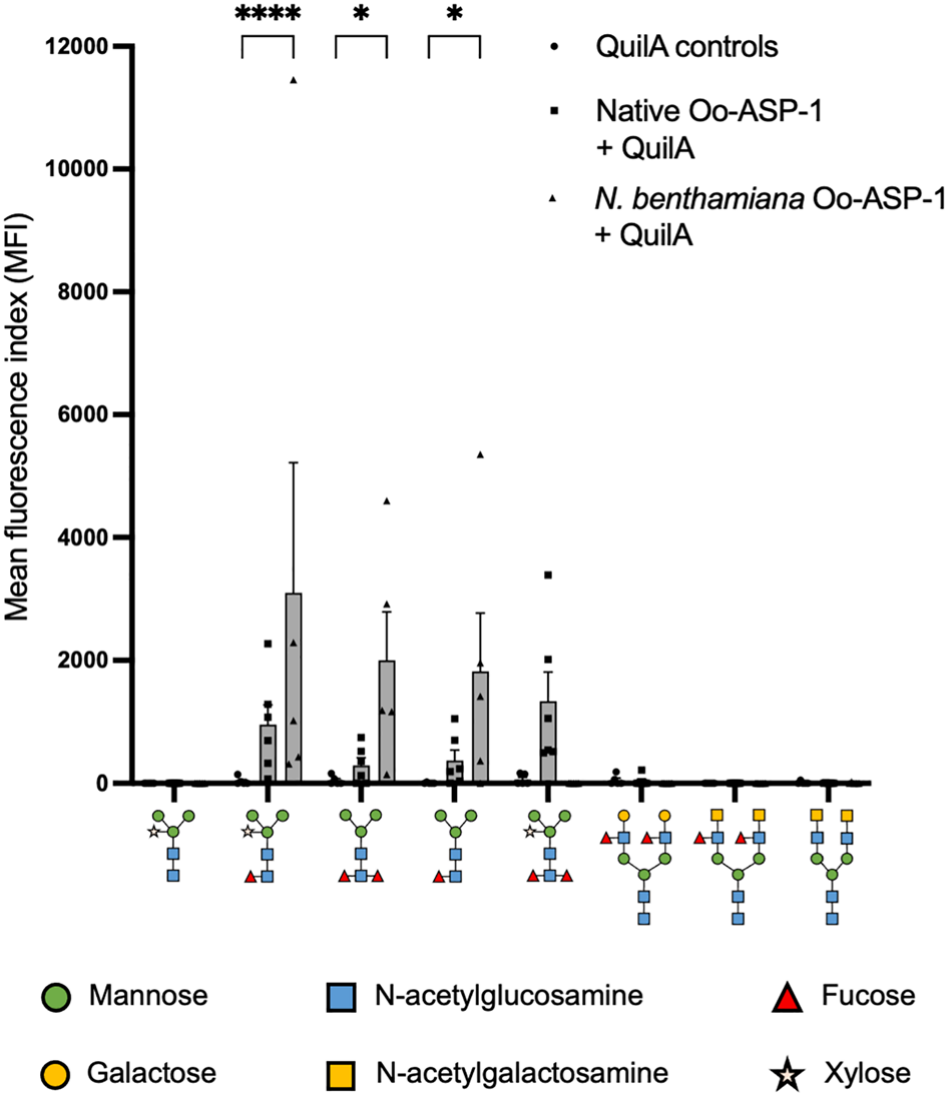
Selective glycan microarray screening. Glycan recognition by native antigen-specific antibodies from calves immunised with either native Oo-ASP-1 + QuilA (n = 6), *N. benthamiana* Oo-ASP-1 + QuilA (n = 5), or QuilA alone (n = 5). Recognition of the glycans displayed on the X-axis is expressed as Mean Fluorescence Index (MFI). Data are presented as mean values + / − standard error of the mean, with the individual data points displayed as symbol. *P* values were calculated via a Two-way ANOVA with Dunnet’s test for multiple comparison. Degrees of freedom for the numerator: 14. Degrees of freedom for the denominator: 104. F distribution value: 2.144. *P < 0.05, **P < 0.01, ***P < 0.001 and ****P < 0.0001 versus QuilA-adjuvant control. Data are representative of two independent experiments. Source data is available upon request.

Finally, to study the impact of introducing the two types of core fucoses on antibody recognition, glycan microarrays were screened with Oo-ASP-1-specific antibodies from calves immunised with native Oo-ASP-1, *N. benthamiana* recombinants or QuilA. Analogous to the native Oo-ASP-1 vaccine group, antibodies from *N. benthamiana*-immunised calves exclusively recognised N-glycans carrying core α1,3-fucose (Fig. 6), but not glycans that carry core α1,6-fucose, or lack fucose overall. Recognition was more defined for *N. benthamiana*-immunised calves compared to native Oo-ASP-1 vaccinated animals, potentially due to the high presence (approximately 50%) of core α1,3-fucose on the *N. benthamiana* recombinants.

## Discussion

The outcome of this study showed that, for the first time, glyco-engineered versions of a nematode vaccine antigen produced in *N. benthamiana* were able to induce a protective immune response against a challenge infection, consistent with the native version of the antigen^11–13^. The contribution of antigen glycosylation in the immune response against helminth infections has been a topic of interest for years. N-glycosylation can contribute in various ways to the increased protective immunogenicity: On a structural level, N-glycans play a crucial role in maintaining the stability of the antigen, aiding in its solubility in aqueous environments^24^, promoting proper protein folding^25^, and protecting against proteolytic activity^26^. Antigen-presenting cells (APC) recognise glycans via specialised pattern-recognition receptors (PRR), such as Toll-like receptors (TLR) and C-type lectin receptors (CLR)^27–29^. These receptors have a regulatory effect on the adaptive immunity, typically by dendritic cell maturation and triggering a Th2 response^30^, and thus play a pivotal role in the early stage of the immune response.

In addition to stimulating innate immune responses, glycans can also be the targets of an antibody response in the infected or immunised host. Several studies have aimed to unravel the role of specific glycan residues on triggering an immune response, as exemplified in the case of *H. contortus* and *Schistosoma mansoni*. It was reported that sheep antibodies raised against core α1,3-fucosylated plant glycoproteins were capable of reacting with α1,3-fucose-carrying glycoproteins from *H. contortus*^31^. For *S. mansoni*, it was demonstrated that IgG1, IgG3, and IgG4 from infected humans displayed a distinctive level of reactivity towards N-glycans that carried core α1,3-fucose^32^. For *O. ostertagi*, glycan array results confirmed the involvement of core α1,3-fucose as part of the antibody epitope, as N-glycans bearing core α1,3-fucose were distinctly recognised by IgGs generated in native Oo-ASP-1 immunised calves. The antibodies were purified against the native antigen, which carries a small fraction of core α1,3-fucose. Therefore, recognition of the α1,3-fucosylated N-glycans is attainable solely through immunisation with α1,3-fucose carrying native Oo-ASP-1 and the *N. benthamiana* recombinants. Calves immunised with *P. pastoris* Oo-ASP-1, lacking this core fucose, induced native Oo-ASP-reactive antibodies that did not demonstrate a clear recognition of N-glycans on the glycan microarray. This observation implies the involvement of core α1,3-fucose as part of an antibody epitope. Interestingly, only core fucose carrying vaccine antigens were able to induce a mucosal IgG response, illustrated in current and previous immunisation trials^11–13^. The exact role of these antibodies in case of *O. ostertagi* remains unclear, but it was demonstrated for sheep that the presence of mucosal antibodies prevented the establishment of *Trichostrongylus colubriformis* in the gut, although such observations were not made for the abomasal nematodes *Teladorsagia circumcincta* and *H. contortus*^33^.

Core α1,6-fucose, the most dominant linkage type of fucose found on native Oo-ASP-1, is generally considered non-immunogenic since it is present on mammalian proteins^34^. However, it is important to note that while certain N-glycan components may not be directly targeted by antibodies, they may still affect the immunogenicity of a protein. For instance, it has been suggested that oligomannosidic glycans present on helminth glycoproteins can bind to several soluble and cell-surface lectin receptors, such as mannose-binding lectin (MBL) and Dendritic Cell-Specific Intercellular adhesion molecule-3-Grabbing Non-integrin (DC-SIGN). Through this process, glycans can facilitate antigen internalisation and modulate the adaptive immune response through lectin-mediated signalling pathways^35^. Depending on its composition, the steric and structural configuration of the N-glycan can positively or negatively impact the accessibility of nearby epitopes, including those associated with the protein backbone^24^.

*N. benthamiana* efficiently addressed the core fucose deficiency observed for *P. pastoris*, resulting in the production of two ASP variants containing either core α1,3-fucose or core α1,6-fucose. The synthesis of recombinant antigens was relatively fast and yielded approximately 1 mg of purified protein per plant, providing sufficient material for extensive in vitro and in vivo assessment. A previous study established that *N. benthamiana* is a suitable expression platform for the production of helminth-type CAP-domain proteins^36^, such as ASPs. It was also shown that the N-glycosylation machinery of the plant is flexible, as previous studies demonstrated the successful synthesis of highly immunogenic GalNAcβ1-4(Fucα1-3)GlcNAc (LDN-F) and Galβ1-4(Fucα1-3)GlcNAc (Lewis X) glycan motifs, as well as core α1,3/α1,6-fucose moieties^37,38^. LDN-F and Lewis X are found on helminths such as *S. mansoni*^39,40^, *H. contortus*^41^, *Opisthorchis viverrine*^42^, *Trichinella spiralis*^43^ and *Trichuris suis*^44^. The flexible nature of this transient expression system could allow for efficient production of diverse glycoproteins with modified N-glycans, which could greatly facilitate the transition towards recombinant subunit vaccines against helminths.

Recombinant ASP-expression was conducted in ΔXT/FT *N. benthamiana* with down-regulated β1,2-xylosyltransferase and α1,3-fucosyltransferase. As previously described, a substantial reduction in the levels of the targeted saccharides was observed, albeit complete elimination was not achieved^45^. This indicates that xylose and core α1,3-fucose were present on all the evaluated ASP-based recombinants, suggesting that the core α1,6-fucose glycovariant also contained a small fraction of α1,3-fucosylated N-glycans, but not vice versa. Consequently, the unavailability of a fucose-lacking version of the *N. benthamiana* recombinant precluded comparative evaluation of antibody recognition and antigen immunogenicity. The *N. benthamiana* recombinants manifested a fraction of glycans carrying xylose, which are not found on native Oo-ASP-1, increasing the heterogeneity of the recombinant’s glycan profile.

Despite the absence of conclusive evidence regarding the importance of terminal galactose on triggering a protective immune response, it was opted to mimic the native N-glycosylation of Oo-ASP-1 to the greatest extent possible, thus by incorporating terminal galactose. After expression in *N. benthamiana*, the presence of terminal galactose on both recombinants was low, attributed to the presence of endogenous beta-galactosidases^46^. Similar observations were made for proteins produced in glyco-engineered *Nicotiana tabacum*^47^, but a higher degree of terminal galactose is expected for *P. pastoris* antigens^48^. Purification with agarose-bound *Ricinus Communis* Agglutinin I (RCA I) raised the ratio of N-glycans with terminal galactose, but N-glycans lacking galactose were still present. As Oo-ASP-1 contains two separate N-glycans, it is likely that lectin-based galactose enrichment occurs even if only one of the N-glycans carries a terminal galactose. Despite the heterogeneity, all dominant hybrid-type N-glycans identified on the native antigen were verified to be present on *N. benthamiana*.

For future studies, reducing the heterogeneity of the N-glycosylation profile and increasing the degree of galactosylation to more closely match the glycans found on the native antigen may positively impact the vaccine-efficacy of the *N. benthamiana* recombinant. Based on the immunogenicity of α1,3-fucose, immunisation with exclusively this glycovariant may further enhance the protective immune response. The rationale in this study for a 1:1 ratio of *N. benthamiana* recombinants carrying core α1,3/α1,6-fucose in both bovine immunisation-infection studies was based on two factors: (1) the documented immunogenicity of core α1,3-fucose and (2) the abundance of core α1,6-fucose on the native Oo-ASP-1 antigen. Evaluating the protective capacity of the α1,3- and α1,6-core fucosylated versions separately would be a next logical step.

To improve the vaccine’s efficacy even further, it may also be required to include recombinant versions of other *O. ostertagi* proteins, such as Oo-ASP-2^49^, cysteine proteinases^50^, gut membrane glycoproteins^51^, *Ostertagia* polyprotein allergen^52^. For *T. circumcincta* in sheep, immunisation with a combination of eight recombinant proteins resulted in a mean reduction in egg output by 45–70%^53,54^. However, it was also stated that the complexity of expression and purification of multiple recombinants negatively impacts a vaccine’s attractiveness for commercialisation^55^. Additional strategies for enhancing vaccine efficacy encompass the assessment of the method of administration, vaccination frequency, antigen dosage per immunisation, and the choice of adjuvants.

*N. benthamiana* proved to be an efficient platform to tailor recombinant protein glycosylation, making it ideal for delivering proof of concept on the importance of N-glycans in antigen immunogenicity. The demonstrated capacity of this system for large-scale vaccine antigen production, as evidenced in the case of COVID-19^56^, holds significant promise for the prospective generation of helminth glycoproteins. To ensure its competitiveness, especially concerning the production of vaccine antigens for the cost-sensitive livestock sector, further optimisation and upscaling of the *N. benthamiana* expression system will be crucial. In addition, the presence of plant-specific glycans remains one of the drawbacks that may hamper industrial confidence^57^.

In terms of the required vaccine efficacy to counter *O. ostertagia* in practice, the observed 39% reduction in cumulative faecal egg output may appear modest. However, obtaining this level of reduction with a recombinant antigen is a unique achievement^58^. In prospect of commercialisation, there are no established guidelines on minimal vaccine efficacy against gastrointestinal nematodes in cattle. In temperate regions with a grazing season of approximately six months, it has been suggested that a 60% decline in the average faecal egg excretion during the first two months of grazing is sufficient to safeguard calves against *O. ostertagi* infections^59^. Consequently, the abovementioned measures are necessary to enhance the efficacy of this recombinant vaccine. For application of the Oo-ASP-1 vaccine, other expression systems should be assessed for their ability to modify N-glycosylation and introduce core fucose, for example the glyco-engineered *P. pastoris* GlycoSwitch strain^60^.

Beyond control of ostertagiosis, this study's findings could pave the way for developing recombinant vaccines against other nematode infections. In the veterinary field, other economically important nematode infections include *Cooperia oncophora* and *Dictyocaulus viviparus* in cattle, and *T. circumcincta* and *H. contortus* in sheep. Immunostimulatory native and recombinant proteins have been identified for these parasites^53,54,61,62^, but transitioning towards a single subunit recombinant vaccine that can be produced on a large scale and provide adequate protection has been challenging.

In conclusion, the comparative analyses conducted between a protective native antigen and a non-protective *P. pastoris* recombinant provided important information on the key elements required to induce a protective immune response. In this study, native Oo-ASP-1 and a non-protective *P. pastoris* recombinant were found to be highly comparable on protein structure, but microarray analysis revealed that native Oo-ASP-1-induced IgG exhibited distinct reactivity towards glycans carrying core α1,3-fucose. This glycan motif was verified to be present on the native antigen but not on the *P. pastoris* recombinant. The employment of glyco-engineered *N. benthamiana* addressed these inconsistencies in N-glycosylation, in particular at the level of core fucosylation, and subsequent immunisation with the new recombinants significantly reduced the faecal egg output in *O. ostertagi* infected calves. These findings are highly promising for the field of recombinant vaccine development, as the transition from native to recombinant subunit vaccines for various parasitic nematodes has been difficult. Therefore, this workflow could be a valuable approach for recombinant vaccine development against other parasitic nematodes.

## Methods

### Production and purification of native and *P. pastoris* Oo-ASP-1

Native Oo-ASP-1 was obtained by collecting adult *O. ostertagi* worms, preparing the ES fraction^63^ and purifying Oo-ASP-1 via thiol-Sepharose^50^ or by applying the ES material to a HiLoad 16/70 Superdex 200 pg column (Cytiva) and performing size exclusion chromatography as described in previous studies^64^. *P. pastoris* recombinant versions of Oo-ASP-1 were expressed and purified as previously described^17^.

### Production and purification of *N. benthamiana* Oo-ASP-1

The ΔXT/FT *N. benthamiana* plants for recombinant expression are a laboratory accession derived from RA-4, and were provided by Dr. Richard Strasser^45^. All handling and use of *N. benthamiana* and associated samples in this study was conducted in compliance with institutional, national, and international guidelines and legislation.

The sequence encoding the mature Oo-ASP-1 protein was codon optimised in-house (Wageningen University and Research) and synthetically constructed at GeneArt. The gene fragment was flanked with NheI/KpnI restriction site for subcloning behind the *Arabidopsis thaliana* chitinase gene (cSP) in the plant expression vector pHYG^37,65^. To engineer α1,6-linked core fucose, the expression vector pBIN-PLUS with *Drosophila melanogaster* fucosyltransferase 8 (DmFUT8) was used^66^. The pHYG vector with *Schistosoma mansoni* fucosyltransferase C (SmFucTC) was used to engineer the α1,3-linked core fucose^38^. The terminal galactose was engineered by expression of a hybrid β1,4-galactosyltransferase (GalT) from *Danio rerio* with a replaced CTS domain of rat α2,6-sialyltransferase under control of the Gpa2 promotor in pHYG^37^. To ensure optimal protein expression, the silencing suppressor P19 from tomato bushy stunt virus in pBIN61 was co-infiltrated. All constructs were transformed to *Agrobacterium tumefaciens* strain MOG101.

Culturing of *A. tumefaciens* (strain MOG101), infiltration of ΔXT/FT transgenic *N. benthamiana* leaves with these bacteria and extraction of the apoplast fluid was done as described previously^37,45^. Apoplast fluid was desalted via Sephadex-G25 columns (Cytiva) and purified using the HS POROS® 50 strong cation exchange resin (ThermoFisher) performed on the ÄKTA Prime Liquid Chromatography System (GE Healthcare). Bound Oo-ASP-1 was eluted from the column using a gradient with cation-exchange buffer supplemented with 1 M NaCl at 2 ml/min, followed by dialysis against phosphate-buffered saline (PBS). Protein concentration was determined using a BCA assay (Pierce, ThermoFisher). Successful expression and purification were confirmed on a 12% Bis–Tris SDS-PAGE gel (ThermoFisher) stained with Coomassie Brilliant Blue.

To increase the level of terminal galactose, each glycoform of the *N. benthamiana* Oo-ASP-1 was subjected to affinity chromatography using agarose-bound *Ricinus communis* agglutinin-I (Vector Laboratories), largely according to the manufacturer’s instructions. Adaptations: Pierce Spin Columns (Thermofisher) were filled with 0.5 ml of the RCA I slurry with 5 column volumes of binding/wash buffer, each time centrifuging at 75 × g for 1 min in order to discard the flowthrough. In 0.5 ml binding/wash buffer, *N. benthamiana* recombinants were applied to the column at a concentration of 1 mg/ml, for an incubation time of 1 h at 4 °C while constantly mixing by inversion. Elution was done by a two-time application of 0.5 ml of Glycoprotein Eluting Solution (Vector Laboratories), followed by 1 min centrifugation at 100 × g.

### Protein evaluation on sodium dodecyl sulfate polyacrylamide gel electrophoresis

All antigens were evaluated on SDS-PAGE under natural conditions, visualised via Coomassie blue staining as mentioned previously^64^. Denaturing SDS-PAGE was carried out including and omitting β-mercaptoethanol in reducing and non-reducing conditions^67^, respectively.

### Oo-ASP-1 RNA extraction, RT-PCR, sub-cloning and sequencing

RNA was extracted from North-American (BARC.NEA, MD, USA) and European (Ghent University, Belgium) 3rd stage larvae separately as follows: after crushing the larvae, the cells were lysed immediately by addition of 1 ml of TRIzol (Gibco, Invitrogen) and incubation at room temperature (RT) for five minutes under constant vortexing. Then, 200 μl of chloroform was added, followed by vigorous shaking for 15 s and incubation on ice for five minutes. After a centrifugation step at 13,500 rpm for ten minutes at 4 °C, the aqueous phase was transferred to a fresh RNase-free tube where the RNA was precipitated by addition of 500 μl of isopropanol and incubation at RT for ten minutes. Centrifugation at 13,500 rpm for ten minutes at 4 °C yielded a pellet which was washed with 1 ml of 75% ethanol and then air-dried. The pellet was subsequently dissolved in 657 μl of diethylpyrocarbonate (DEPC) and DNase treated (addition of 9 μl RNase inhibitor and 9 μl DNase I) with incubation at 37 °C for 30 min. After addition of 750 μl of chloroform and vigorous shaking for 15 s, the sample was centrifuged at 13,500 rpm for ten minutes at 4 °C. The aqueous phase was then transferred to a fresh RNase-free tube and the chloroform-addition step was repeated. The aqueous phase was again transferred to a fresh RNase-free tube where the RNA was precipitated by adding 500 μl of isopropanol with incubation of the sample at RT for ten minutes. After centrifugation of the sample at 13,500 rpm for ten minutes at 4 °C the supernatant was removed and the pellet was washed thoroughly by adding 900 μl ethanol. A centrifugation step at 13,500 rpm for five minutes at 4 °C followed, with subsequent removal of the supernatant and air-drying of the pellet at RT for about ten minutes. The pellet was dissolved in 15 to 25 μl of DEPC water and the RNA concentration was determined. RNA was then converted into cDNA via reverse-transcriptase polymerase chain reaction (RT-PCR) according to the SuperScript™ One-Step RT-PCR with Platinum®*Taq* System protocol (Invitrogen). PCR mixtures were cleaned up using the GENECLEAN® kit (Q-BioGene) after which purified PCR product was ligated into the pGEM®-T Easy vector (Promega) according to the manufacturer’s instructions. PCR mixtures typically consisted of 50 ng RNA, 0.4 μM of both forward and reverse primers (ASP1-F: ATGCAGGCACTAATCGGTATTGCT and ASP1-R: ATCCGAGTCGATTTACAAGGCTGG, respectively) and one unit of RT/Platinum® *Taq* Mix in reaction buffer containing 0.2 mM of each dNTP and 1.2 mM MgCl_2_. PCR experiments were carried out on a Mastercycler® Ep instrument (Eppendorf) with program settings as follows: after an initial 30 min at 50 °C a denaturing temperature of 94 °C is maintained for two minutes, followed by 32 cycles of denaturing (15 s at 94 °C), annealing (30 s at 60 °C) and elongation (DNA synthesis for 45 s at 72 °C). Afterwards, a final elongation step at 72 °C for ten minutes was included, after which the PCR mixtures were kept at 10 °C. Prior to ligation, a clean-up of the PCR mixtures was carried out using the GENECLEAN® kit (Q-BioGene) after which purified PCR product was ligated into the pGEM®-T Easy vector (Promega) according to the manufacturer’s instructions. Subsequently, the ligation mixtures were transformed in *Escherichia coli* DH5α competent cells (Invitrogen) according to the supplier’s instructions. After overnight incubation at 37 °C on X-gal plates, white colonies were picked from both the North-American and European plates. On all of the picked colonies PCR was performed using the same primers and reagent concentrations as mentioned above, and the program settings were as follows: after an initial denaturing temperature of 95 °C for two minutes, 32 cycles of denaturing (30 s at 95 °C), annealing (30 s at 60 °C) and elongation (DNA synthesis for 50 s at 72 °C) were carried out. Afterwards, a final elongation step at 72 °C for five minutes was included, after which the PCR mixtures were all bidirectionally sequenced.

### Circular dichroism spectroscopy

CD data were recorded on a J-715 spectropolarimeter (JASCO) equipped with a 0.1 cm path length quartz cuvette (Hellma Analytics) and applying native Oo-ASP-1 and *P. pastoris* Oo-ASP-1 at 10 μM in PBS. Ellipticity was monitored from 260 to 190 nm, with 0.5 nm intervals.

### Liquid chromatography coupled to mass spectrometry

Tertiary and quaternary structure insights, focussing on intra- and intermolecular disulphide bonds in the studied Oo-ASP-1 molecules, were obtained via one-dimensional and two-dimensional LC–MS (liquid chromatography coupled to mass spectrometry), thereby using an Agilent 6230B hybrid-Q-TOF mass spectrometer (Agilent Technologies) with MS and MS/MS scan ranges set to 300–3000 and 100–3000 Th, respectively, and acquisition speed at one spectrum per second. Briefly, 50 μg of the sample under investigation was supplemented with 0.1% (v/v) Rapigest (Waters) in 100 mM Tris–HCl pH 8.0, followed by treatment with 5 mM DTT (dithiotreitol) and 10 mM iodoacetamide (IAA) when reduction of dithioether bonds was required, and addition of MS-grade trypsin (Promega) to a final concentration of 0.02 μg per μg protein. For the two-dimensional approach, a reversed-phase LC (RPLC) step was performed at pH 8.0, using a C18 column (Agilent Technologies) held at 50 °C with 0.1% formic acid in water as the primary solvent and a gradient of 1.5% acetonitrile per minute as the eluting solvent (flow-rate was 200 μl/min.), with fraction collection every 30 s prior to injection in the mass spectrometer. Comparison between the one- and two-dimensional data of reduced and non-reduced samples allowed mapping of the individual cysteine (Cys) residues being part of specific intra- or intermolecular disulphide bridges.

### N-glycan profiling via MS and identification of core fucose linkage

The N-glycosylation of native, *P. pastoris* and *N. benthamiana* Oo-ASP-1 was evaluated via MS after PNGase A release as described in a previous study^37^. Specific for *N. benthamiana*, MS was conducted before and after RCA I affinity purification.

To confirm the presence of core α1,3-fucose on native Oo-ASP-1, treatment of the released N-glycans with hydrogen fluoride (HF) was conducted as described previously^68^, only removing core α1,3-linked fucose. This was followed by MS analysis as described above. For *N. benthamiana* recombinants, the presence of the specific core fucose linkages was analysed via lectin assays. Biotinylated *Aleuria aurantia* lectin (AAL) (Vector Laboratories) was used to determine core fucosylation in general, whilst biotinylated *Pholiota squarrosa* lectin (PhoSL) (kindly provided by Dr. A. Varrot from Université Grenoble Alpes, Grenoble, France) was used for core α1,6-fucose specifically. Microtiter plates were coated overnight at 4 °C with purified *N. benthamiana* Oo-ASP-1 in PBS at a concentration of 1 or 10 µg/ml. The lectin binding assay was performed as previously described^38^.

### Glycan microarrays screening with Oo-ASP-1 specific IgG

Bovine serum samples were obtained from calves immunised with either native Oo-ASP-1 + QuilA, *P. pastoris* Oo-ASP + QuilA, or QuilA alone, one week after the second immunisation. ASP-specific antibodies were isolated in order to prevent false positives and a high background fluorescence due to glycan recognition by non-ASP-specific antibodies. To do this, equal volumes of pooled serum samples (n = 11) were first subjected to lipoproteins and lipids removal via the addition of dextran sulphate and calcium chloride, followed by centrifugation. Protein G HP SpinTrap columns (Cytiva) were utilised to purify immunoglobulins (Ig’s), according to the manufacturer’s protocol. The ASP-specific antibodies were subsequently purified via NHS HP SpinTrap colums (Cytiva) coupled with native Oo-ASP-1 antigens, according to the manufacturer’s protocol. After purification, all samples were dialysed repeatedly with Slide-A-Lyzer MINI dialysis devices (ThermoFischer) in order to obtain a PBS-buffered solution and evaluated for ASP-reactivity through ELISA/Western Blot.

Non-commercial synthetic microarray slides were constructed as previously described^22^. The binding assay with antibodies mentioned above was performed as described in a previous study^69^ with the following deviations: purified antibodies were tested at 1/500 in PBS-0.01% Tween20 with 1% BSA, followed by an incubation with 1/1000 Cy3-conjugated goat anti-bovine IgG (Jackson Laboratories) in PBS-0.01% Tween20. The mean fluorescence index (MFI) was obtained via the G2565BA scanner (Agilent Technologies) and was analysed with GenePix Pro 7.0 software (Molecular Devices). The MFI for each N-glycan spot was exported to Microsoft Excel where the background MFI was subtracted for each glycan structure.

To study the reactivity of ASP-specific antibodies from *N. benthamiana* immunised calves towards α1,3-fucose carrying N-glycans, new arrays were generated as described previously^68^. The antibody binding assay was conducted as described above.

### Indirect and competition enzyme-linked immunosorbent assay

Indirect ELISAs were performed as described previously^13^. Slight modifications were made as 96-well ELISA plates (MaxiSorp, NUNC) were coated with 1 µg/ml native Oo-ASP-1 in 100 µl carbonate buffer (pH 9.6) overnight at 4 °C. Serum samples and HRP-conjugated antibodies were used at following concentrations: bovine serum at 1/200 in PBS, sheep anti-bovine IgG1-HRP (AAI21P; Bio-Rad) at 1/4000 in blocking buffer (2% bovine serum albumin in PBS-0.05% Tween20), sheep anti-bovine IgG2-HRP (AAI22P; Bio-Rad) at 1/1000 in blocking buffer, and 1/1000 anti-bovine IgA-HRP (AAI49P; Bio-Rad) at 1/1000 in blocking buffer.

For competition ELISAs, 96-well Maxisorp plates (MaxiSorp, NUNC) were coated with 1 µg/ml native Oo-ASP-1 and dilution series of test antigens ranging from 0 to 500 pmol/ml were generated as described previously^13^. A 1/4000 sheep anti-bovine IgG1-HRP (AAI21P; Bio-Rad) was used to monitor antibody binding against the IgG1 isotype. 2,2′-azino-di-(3-ethylbenzthiazoline sulfonic acid) (ABTS) (Roche) was used as substrate and the colour development, expressed as OD405-492, was quantified by using an Infinite F50 Absorbance Microplate Reader (Tecan Trading AG).

### Bovine immunisation experiment

All animal experiments were conducted in accordance with the E.U. Animal Welfare Directives and VICH Guidelines for Good Clinical Practice, and ethical approval to conduct the studies were obtained from the Ethical Committee of the Faculty of Veterinary Medicine, Ghent University (EC2018/001, EC2020/089, EC 2022/005). The animal experiments were carried out in compliance with the ARRIVE guidelines.

In total, three bovine immunisation studies were conducted with various Oo-ASP-1 antigens. For the first study, aiming to confirm the protective capacity of size-exclusion purified native Oo-ASP-1, fourteen helminth naïve male Holstein calves of 4–6 months old were randomised over two groups of seven animals: native Oo-ASP-1 + QuilA or QuilA alone. The second study, evaluating *N. benthamiana* Oo-ASP-1, contained twenty-four male Holstein calves of 4–6 months old, randomly divided over three vaccine groups of eight animals: native Oo-ASP-1 + QuilA, *N. benthamiana* Oo-ASP-1 + QuilA group, or QuilA alone. For the third study, a repeated trial with *N. benthamiana* Oo-ASP-1, twenty-four male Holstein calves of 4–6 months old were randomly assigned to two vaccine groups of twelve animals: *N. benthamiana* Oo-ASP-1 + QuilA or QuilA alone. For both trials, a 1:1 mixture of core α1,3-fucose / core α1,6-fucose containing *N. benthamiana* Oo-ASP-1 was used.

The trials were conducted as described previously^12,13,50^. In short, all animals received 30 µg of antigen and/or 750 µg of QuilA percutaneous in the neck muscle, three times with a three-week interval. Immediately after the third immunisation, all animals were challenged via a trickle infection of 25,000 *O. ostertagi* L3 (1000 L3 / day; 5 days/week; for 5 weeks). All calves were euthanised three weeks after the last infection with intravenous injection of pentobarbital sodium (450–900 mg/10 kg body weight). Faecal egg counts, worm counts and worm measurements were conducted as described in previous trials^12,50^. For the third trial, faecal egg counts were performed with MiniFLOTAC^70^, instead of McMaster. Serum samples were collected prior to immunisation and one week after each immunisation. Mucus samples were collected and processed as described in previous trials^13^.

### Statistical analyses

The statistical analyses were performed in GraphPad Prism 9. For the bovine immunisation-challenge study with size-exclusion purified native Oo-ASP-1 versus QuilA controls, cumulative faecal egg output and worm counts were evaluated for statistical significance via one-tailed Mann–Whitney tests. For the bovine immunisation-challenge study with native Oo-ASP-1, *N. benthamiana* Oo-ASP-1 and QuilA controls, cumulative faecal egg output, worm counts and worm measurements were evaluated for statistical significance via Kruskal–Wallis test with Dunn’s test for multiple comparison. The systemic IgG1 and IgG2 response was evaluated for all timepoints via a Two-way ANOVA with Dunnet’s test for multiple comparison. The mucosal IgG1, IgG2 and IgA response was evaluated via Kruskal–Wallis tests with Dunn’s test for multiple comparison. For the bovine immunisation-challenge study with *N. benthamiana* Oo-ASP-1 versus QuilA controls, cumulative faecal egg output, worm counts and worm measurements were evaluated for statistical significance via one-tailed Mann–Whitney tests. The systemic IgG1 and IgG2 response was evaluated for all timepoints via a two-way ANOVA with Dunnet’s test for multiple comparison. The mucosal IgG1, IgG2 and IgA response was evaluated via one-tailed Mann–Whitney tests. Glycan microarrays with native ASP-specific antibodies from calves immunised with either native Oo-ASP-1, *P. pastoris* Oo-ASP-1, *N. benthamiana* Oo-ASP-1 or QuilA was evaluated for statistical significance via a two-way ANOVA with Dunnet’s test for multiple comparison. All data were presented as mean ± standard error of the mean (SEM). *P* < 0.05 was considered significant.

### Informed consent

Information concerning the *N. benthamiana* plants that were used for the recombinant expression of vaccine antigens used in this study: The origin of the widely distributed *N. benthamiana* laboratory accession RA-4 has recently been described by Wylie and Li (https://doi.org/10.3390/v14040771). Leaf material and seed was obtained from one of the *N. benthamiana* plants John Cleland collected at The Granites on the 25 August 1936, hereafter referred to as *N. benthamiana* Granites. This specimen was lodged in the Australian National Herbarium in Canberra by Cleland, and its origin is recorded as *The Granites, 400 miles NW of Alice Springs, coordinates − 20.5667, 103.35, Tanami Region of the Northern Territory.* The catalogue number is CANB112241.1

### Supplementary Information

Supplementary Information.

## Data Availability

The datasets generated during and/or analysed during the current study are available from the corresponding author on reasonable request. NCBI GenBank accession number for the sequence of Oo-ASP-1: AJ310812.2. NCBI GenBank accession numbers for the sequences of the Oo-ASP-1 allelic variants: OR750503-OR750509.
