# Supplementary material for: Plant-based production of a protective vaccine antigen against the bovine parasitic nematode Ostertagia ostertagi
Source: Sci Rep. 2023 Nov 22;13:20488. doi: 10.1038/s41598-023-47480-3 (PMC10665551; doi:10.1038/s41598-023-47480-3)
Supplement: Supplementary file 1 — Supplementary Information. [file 41598_2023_47480_MOESM1_ESM.pdf]

Plant-based production of a protective vaccine antigen against the bovine parasitic nematode *Ostertagia ostertagi*.

Laurens Zwanenburg[1], Jimmy Borloo[1], Bregt Decorte[1], Myrna J.M. Bunte[2], Sanaz Mokhtari[2], Sonia Serna[4], Niels-C. Reichardt[4], Leen J.M. Seys[1], Angela van Diepen[3], Arjen Schots[2], Ruud H.P. Wilbers[2], Cornelis H. Hokke[3], Edwin Claerebout[1], Peter Geldhof[1,*].

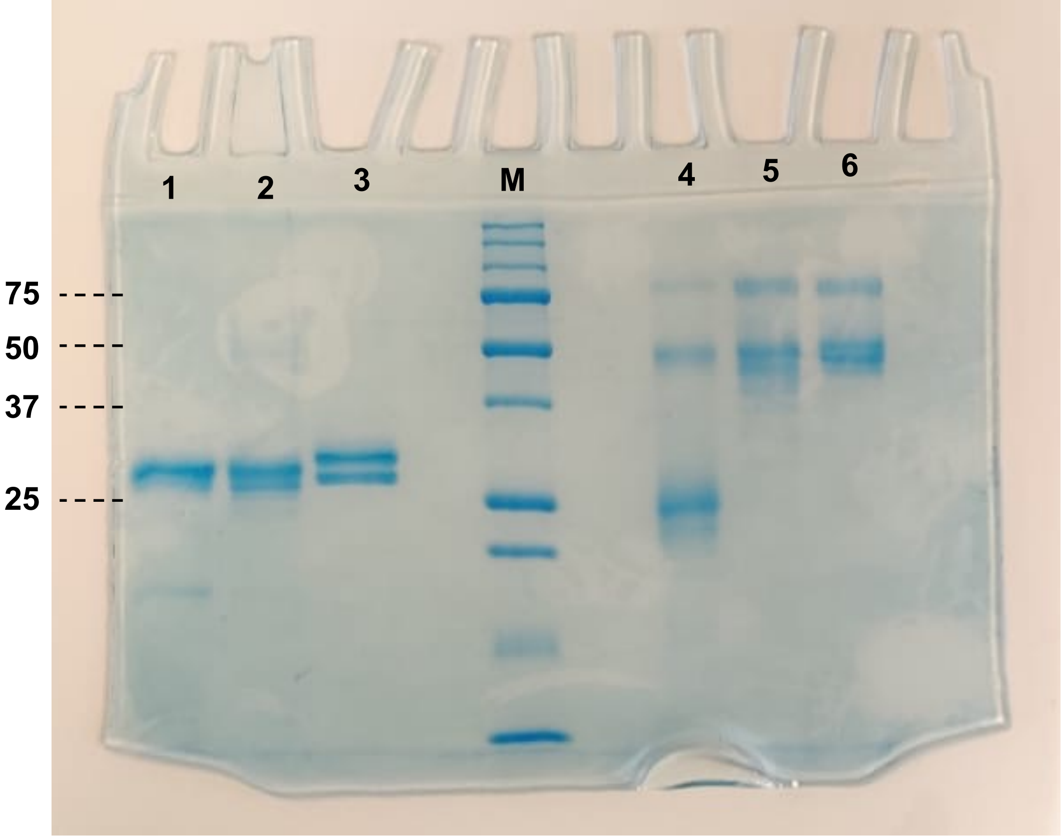

Supplementary Figure 1. Native and recombinant Oo-ASP-1 dimerisation. SDS-PAGE analysis showing the migration patterns for Precision Plus ProteinTM standard (M), thiol-Sepharose purified Oo-ASP-1 (lane 1, reducing; lane 4, non-reducing), size-exclusion purified Oo-ASP-1 (lane 2, reducing; lane 5, non-reducing) and Pichia pastoris recombinant Oo-ASP-1 (lane 3, reducing; lane 6, non-reducing). Molecular weight of 25, 37, 50 and 75 kDa protein bands are displayed on the left side of the figure. Presented data is based on two separate experiments. Source data is available upon request.

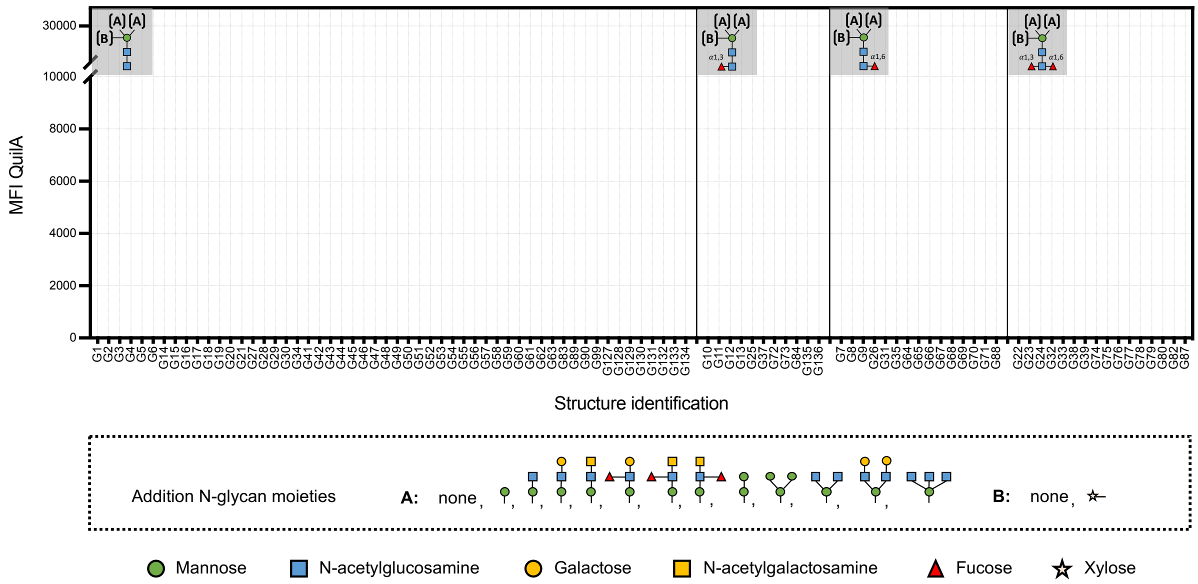

Supplementary Figure 2. Glycan microarray with native ASP-reactive antibodies from QuilA-immunised calves. Glycan recognition by pooled (n=11) native antigen-reactive antibodies from QuilA immunised calves, expressed as Mean Fluorescence Index (MFI). The X-axis contains the structure identification codes for the glycans, as previously published[22]. Glycan structures are organised based on core fucosylation type, depicted in the top-left corner of each frame. Further modifications are indicated by "*A*" and "*B*", and are displayed in the legend below. Data are presented as mean values +/− standard error of the mean of four technical replicates. Presented data is based on a single experiment. Source data is available upon request.

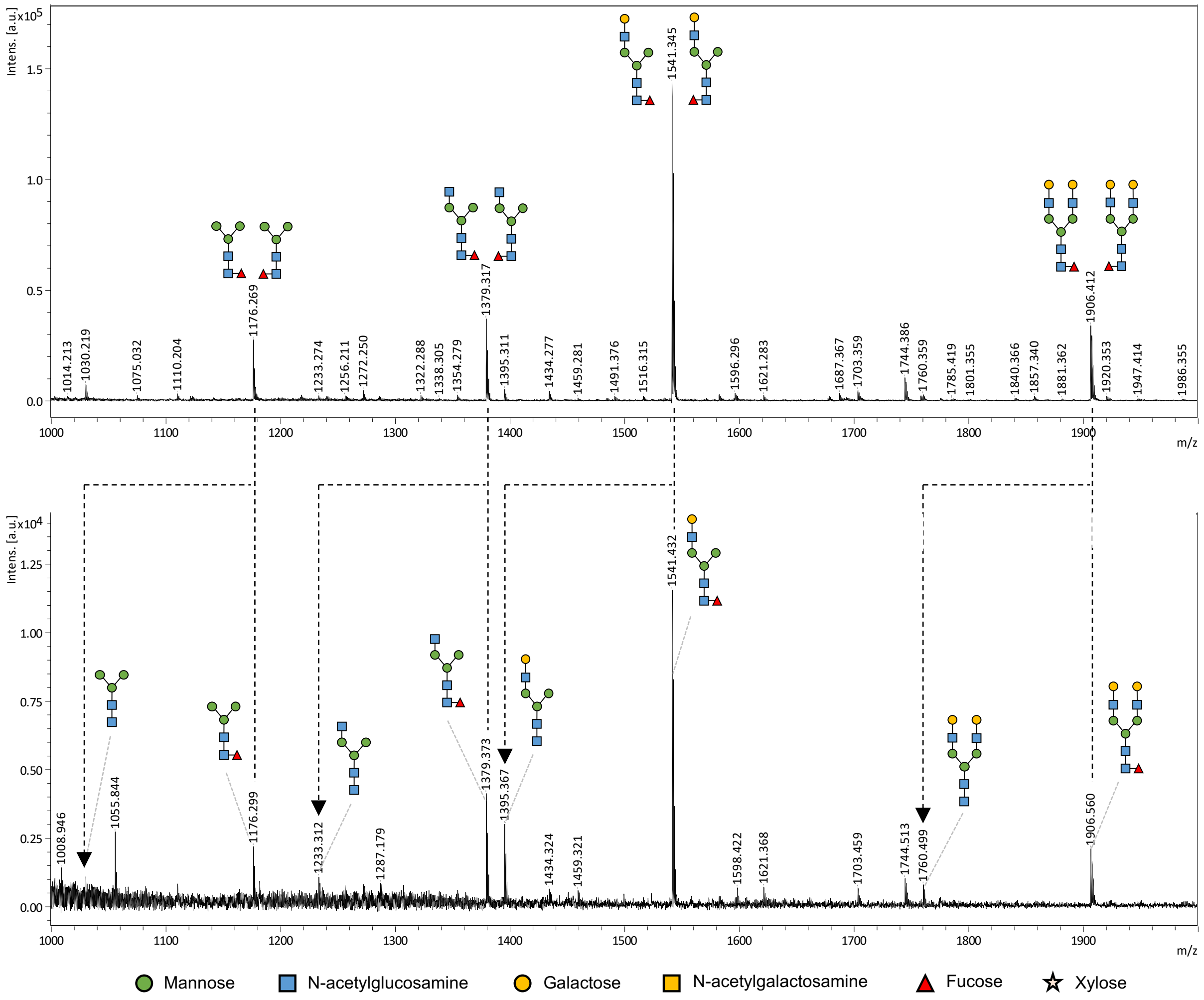

Supplementary Figure 3. Confirmation core α1,3-fucose on native Oo-ASP-1. Mass-spectrometry analysis of the N-glycosylation profile of native Oo-ASP-1 before (upper panel) and after (lower panel) hydrofluoric acid (HF) treatment, removing only α1,3-linked fucose. Shift in mass due to HF treatment is indicated by arrows. Monoisotopic masses of measured signals are indicated, and the proposed glycan structures are depicted based on the Consortium for Functional Glycomics (CFG) nomenclature. The X-axis displays the mass to charge ratio (m/z), whilst the Y-axis displays the relative intensity in arbitrary units (intens. [a.u.]). Presented data is based on a single experiment. Source data is available upon request.

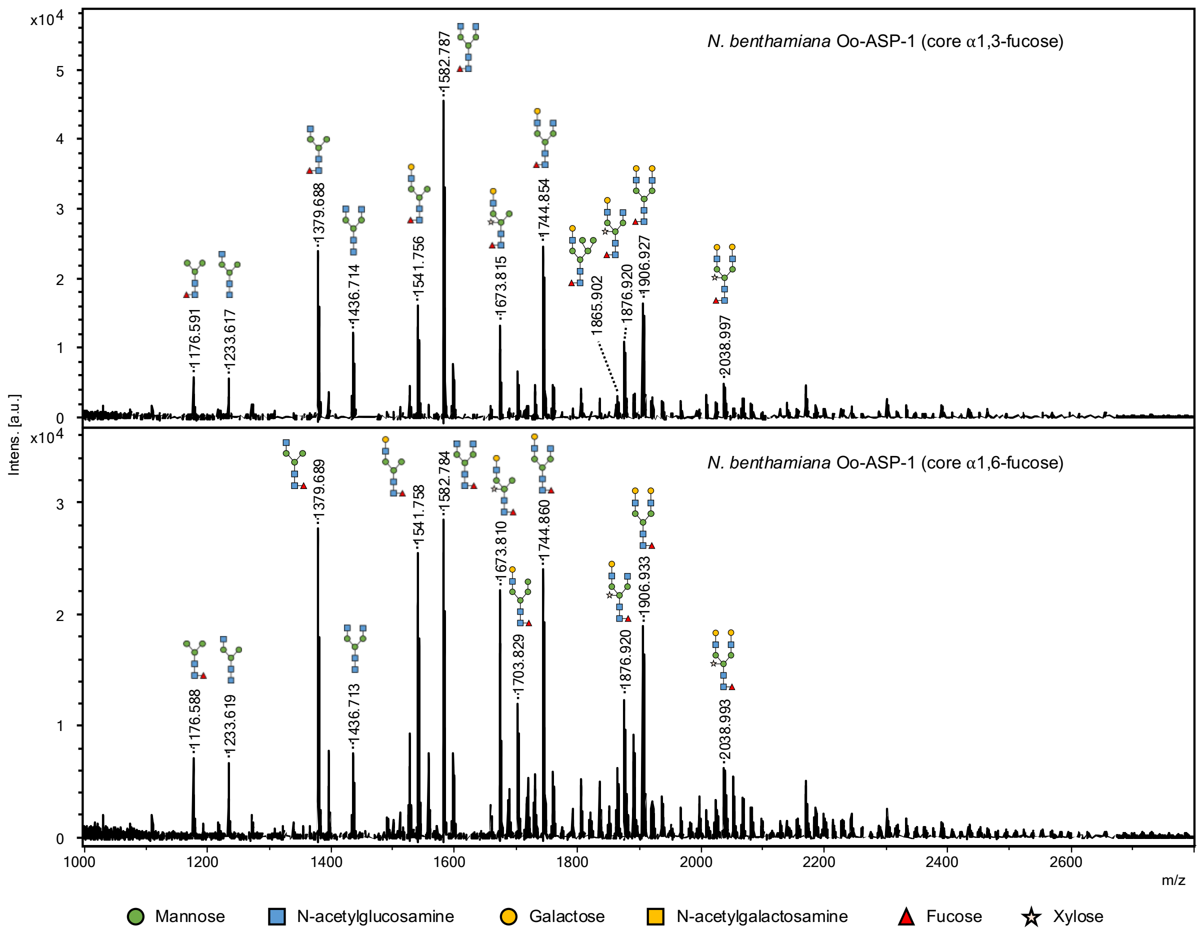

Supplementary Figure 4. *Nicotiana benthamiana* Oo-ASP-1 terminal galactose enrichment. Mass-spectrometry analysis of the N-glycosylation profile of *Nicotiana benthamiana* Oo-ASP-1 with core α1,3-fucose (upper panel) and core α1,6-fucose (lower panel) after enrichment for terminal galactose via agarose-bound *Ricinus communis* agglutinin I (RCA I). Monoisotopic masses of measured signals are indicated, and the proposed glycan structures are depicted based on the Consortium for Functional Glycomics (CFG) nomenclature. The X-axis displays the mass to charge ratio (m/z), whilst the Y-axis displays the relative intensity in arbitrary units (intens. [a.u.]). Presented data is based on a single experiment. Source data is available upon request.

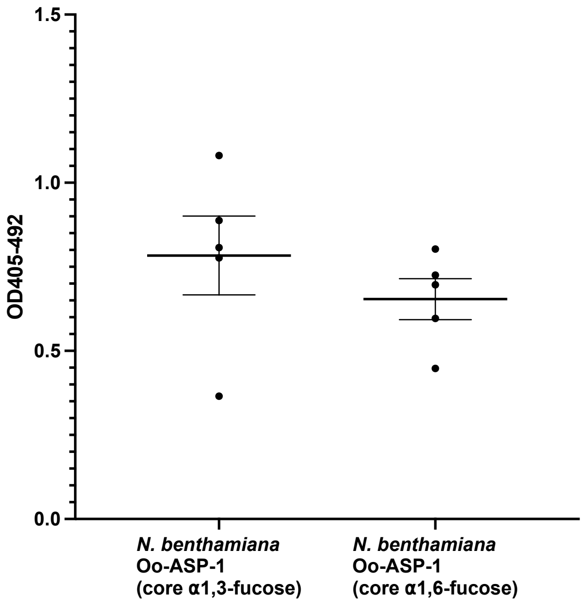

Supplementary Figure 5. Indirect ELISA on *Nicotiana benthamiana* Oo-ASP-1. Recognition of *Nicotiana benthamiana Oo*-ASP-1 with either core α1,3-fucose or core α1,6-fucose by serum samples (n=5) from calves immunised with native Oo-ASP-1, expressed as optical density (OD) measured 405 nm with 492 nm background subtracted. Data are presented as mean values +/− standard error of the mean. Presented data is based on a single experiment. Source data is available upon request.

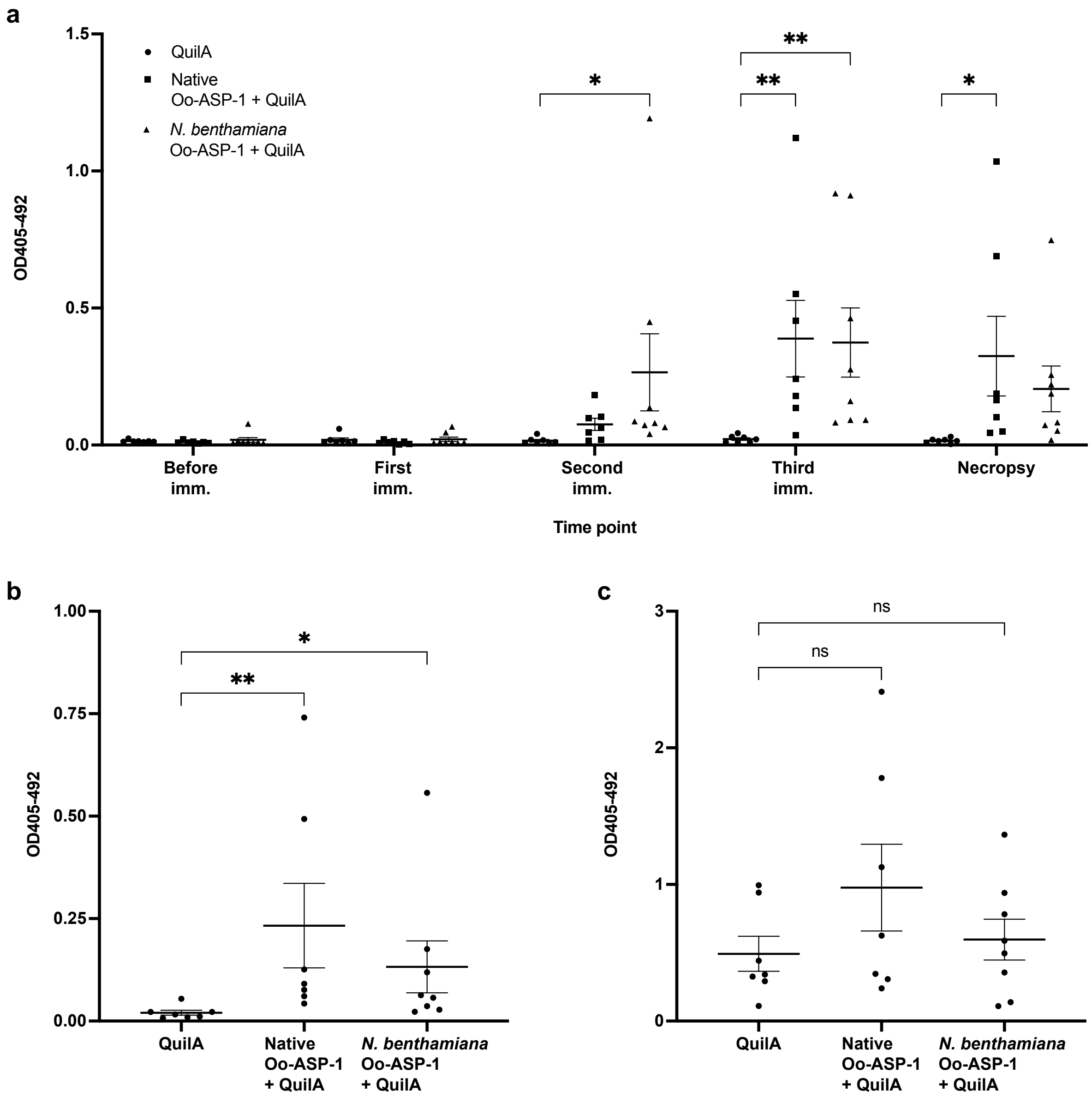

Supplementary Figure 6. Systemic and local IgG2/IgA response in calves. Calves were immunised three times with either native Oo-ASP-1 + QuilA (n=7), *Nicotiana benthamiana* Oo-ASP-1 + QuilA (n=8) or QuilA alone (n=7) prior to a trickle infection with L3-stage *Ostertagi ostertagi*. *(a)* The systemic IgG2 response to native Oo-ASP-1 in an ELISA was measured prior to immunisation (imm), one week after each immunisation and at necropsy, displayed in optical density 405 nm with background correction at 492 nm. *(b)* The mucosal IgG2 and *(c)* mucosal IgA response to native *Oo*-ASP-1 was measured at time of necropsy, displayed in optical density 405 nm with background correction at 492 nm. Data are presented as mean values +/− standard error of the mean. P values for (a) were calculated using a Two-way ANOVA with Dunnet's test for multiple comparison. Degrees of freedom for the numerator: 8.

Degrees of freedom for the denominator: 95. F distribution value: 1.89. P values for (b) and (c) were calculated using a Kruskal-Wallis test with Dunn's test for multiple comparison. *$P < 0.05$, **$P < 0.01$, ***$P < 0.001$ and ****$P < 0.0001$ versus QuilA-adjuvant control. The experiments for all figures were conducted once. Source data is available upon request.

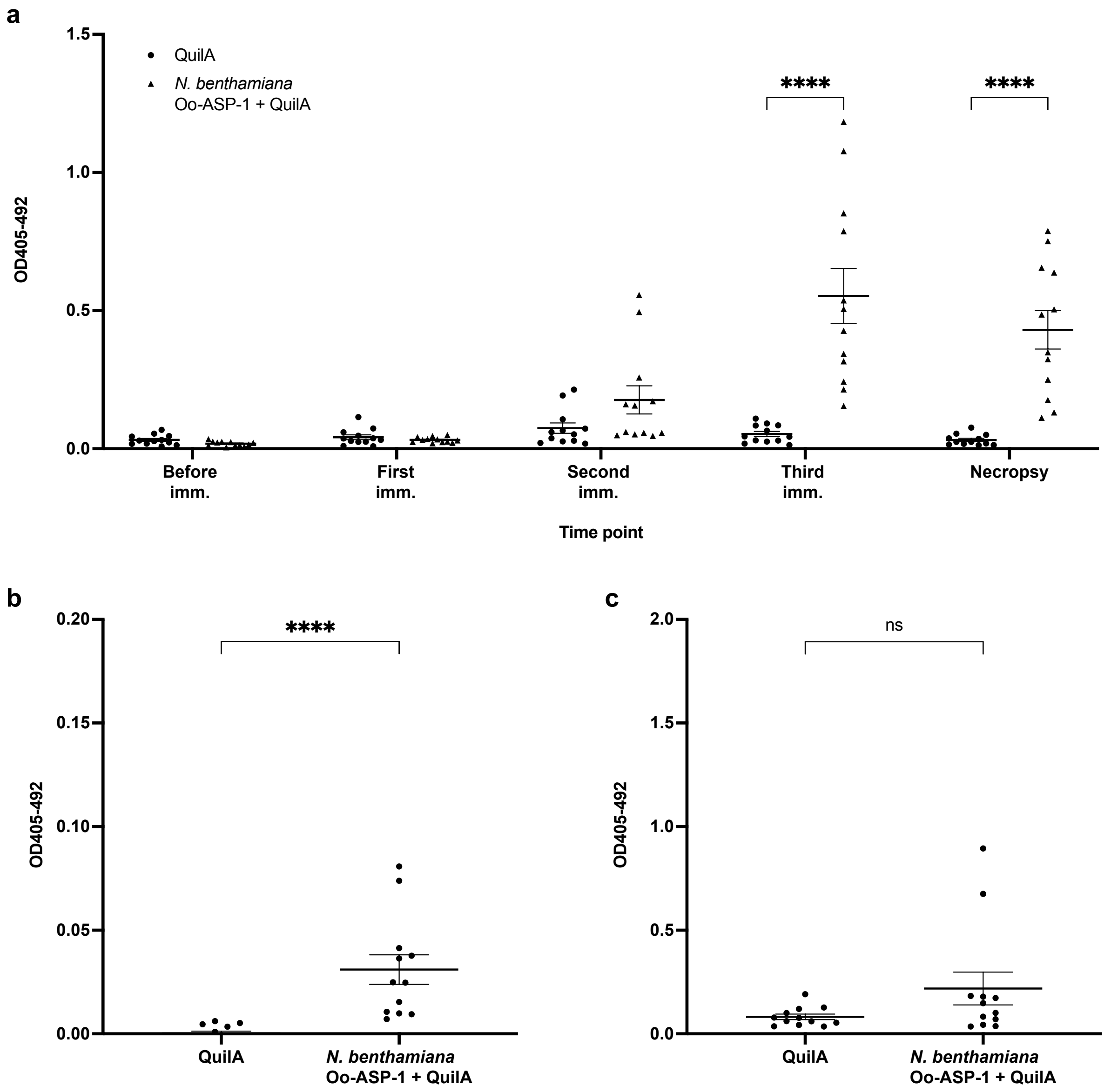

Supplementary Figure 7. Systemic and local IgG2/IgA response in calves. Calves were immunised three times with either *Nicotiana benthamiana* Oo-ASP-1 + QuilA (n=12) or QuilA alone (n=12) prior to a trickle infection with L3-stage *Ostertagi ostertagi*. *(a)* The systemic IgG2 response to native Oo-ASP-1 in an ELISA was measured prior to immunisation (imm), one week after each immunisation and at necropsy, displayed in optical density 405 nm with background correction at 492 nm. *(b)* The mucosal IgG2 and *(c)* mucosal IgA response to native Oo-ASP-1 was measured at time of necropsy, displayed in optical density 405 nm with background correction at 492 nm. Data are presented as mean values +/− standard error of the mean. P values for (a) were calculated using a Two-way ANOVA with Dunnet's test for multiple comparison. Degrees of freedom for the numerator: 4. Degrees of freedom

for the denominator: 110. F distribution value: 15.96. P values for (b) and (c) were calculated using a one-tailed Mann-Whitney test. *P < 0.05, **P < 0.01, ***P < 0.001 and ****P < 0.0001 versus QuilA-adjuvant control. The experiments for all figures were conducted once. Source data is available upon request.

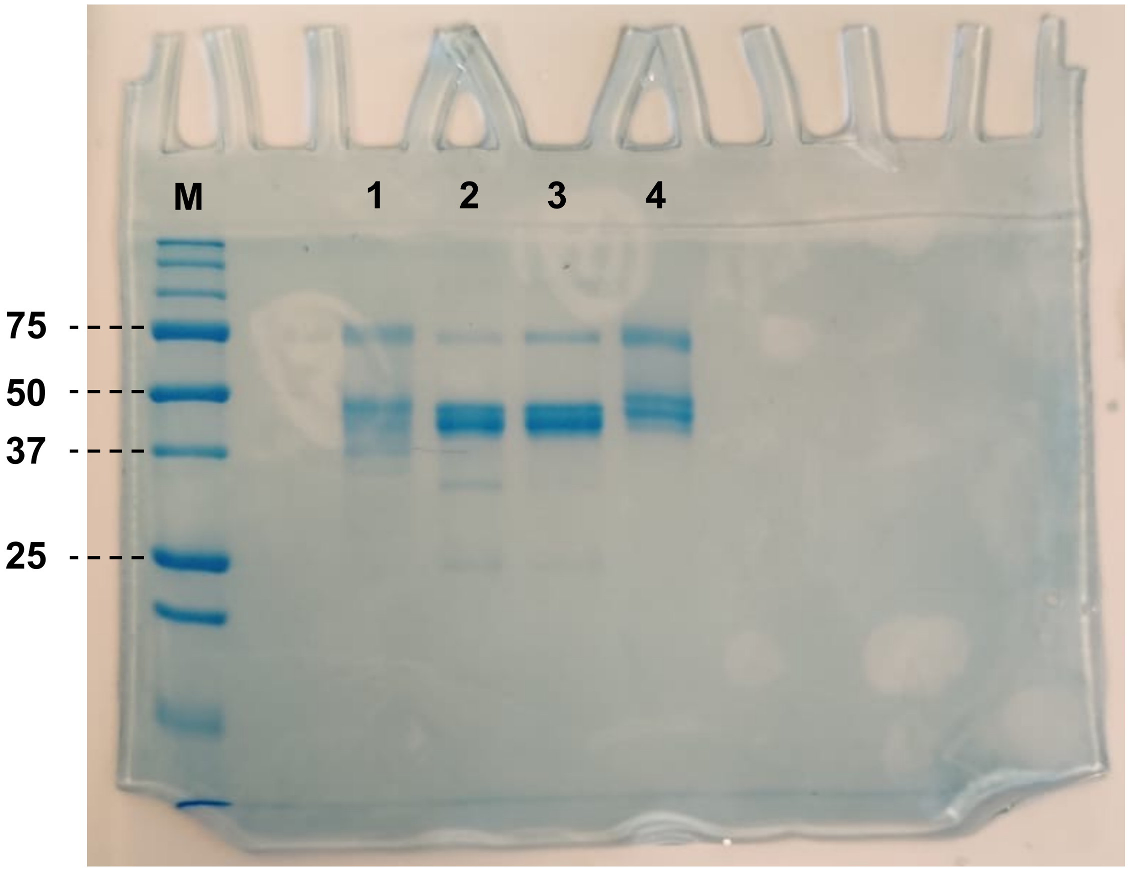

Supplementary Figure 8. Uncropped SDS-PAGE with native and recombinant Oo-ASP-1. SDS-PAGE under non-reducing conditions showing the migration patterns for Precision Plus Protein^{TM} standard (M), native Oo-ASP-1 (lane 1), *N. benthamiana* Oo-ASP-1 with core α1,3-fucose (lane 2), *N. benthamiana* Oo-ASP-1 with core α1,6-fucose (lane 3) and *P. pastoris* Oo-ASP-1 (lane 4). All antigens were applied twice to this SDS-PAGE, hence a repetition of the numbers on the left and right side of the figure. Molecular weight of 25, 37, 50 and 75 kDa protein bands are displayed on the left side of the figure. No contrast adjustments were made. Presented data is based on two independent experiments. Source data is available upon request.

Peptides	Mass	Disulphide bond #	Free thiol
**Thiol-Sepharose purified native Oo-ASP-1**			
VGCAHK + FPTGTNMVVSCAYGGEVLQDNEVVWQK	3581.674	3	no
GPTCECNAYPNSFCCNNLCDTIAAATLR + GPTCECNAYPNSFCCNNLCDTIAAATLR	5918.389	4, 5, D	no
NQPCKST	776.345	6	yes
**Size-exclusion purified native Oo-ASP-1**			
DFCCPADLNQTDEAR + MDWDCNLEAK + NQPCK	3504.386	1, 6	no
GFCCPADLNQTDEAR + MDWDCNLEAK + NQPCK	3446.398	1, 6	no
VGCAHK + VCKFPTGTNMVVSCAYGGEVLQDNEVVWQK + AMIWPCTTPLPIDTSIPQNLAQWLLFQNSQENEVLTQTPWSWVTASLR	9433.551	2, 3	no
VGCAHK + VCKFPTGTNMVVSCAYGGEVLQDNEVVWQK + AMIWPCTTPLPIDTSIPQNLAQWLLFQNSQETEVLTQTPWSWVTASLR	9420.523	2, 3	no
VGCAHK + VCKFPTGTNMVVSCAYGGEVLQDNEVVWDK + AMIWPCTTPLPIDTSIPQNLAQWLLFQNSQENEVLTQTPWSWVTASLR	9420.523	2, 3	no
VGCAHK + VCKFPTGTNMVVSCAYGGDVLQDNEVVWQK + AMIWPCTTPLPIDTSIPQNLAQWLLFQNSQESEVLTQTPWSWVTASLR	9392.501	2, 3	no
VGCAHK + VCKFPTGTNMVVSCAYGGEVLQDNEVVWQK + AMIWPCTTPLPIDTSIPQNLAQWLLFQNSQESEVLTQTPWSWVTASLR	9406.528	2, 3	no
VGCAHK + VCKFPTGTNMVVSCAYGGDVLQDNEVVWQK + AMIWPCTTPLPIDTSIPQNLAQWLLFQNSQETEVLTQTPWSWVTASLR	9406.528	2, 3	no
VGCAHK + FPTGTNMVVSCAYGGEVLQDNEVVWQK	3581.659	3	no
VGCAHK + FPTGTNMVVSCAYGGEVLQDNEVVWDK	3568.629	3	no
AMIWPCTTPLPIDTSIPQNLAQWLLFQNSQENEVLTQTPWSWVTASLR + VCK	5869.903	2	no
AMIWPCTTPLPIDTSIPQNLAQWLLFQNSQESEVLTQTPWSWVTASLR + VCK	5842.894	2	no
AMIWPCTTPLPIDTSIPQNLAQWLLFQNSQETEVLTQTPWSWVTASLR + VCK	5856.908	2	no
AMIWPCTTPLPIDTSIPQNLAQWLLFQNSQENEVLTQTPWSWVTASLRNLQPDTEANIYNWQIRPL SNIANWQNLK + VCK	9204.577	2	no
GPTCECNAYPNSFCCNNLCDTIAAATLR + GPTCECNAYPNSFCCNNLCDTIAAATLR	5918.356	4, 5, D	no
GPTCMCNAYPNSFCCNNLCDTIAAATLR + GPTCECNAYPNSFCCNNLCDTIAAATLR	5920.356	4, 5, D	no
GPTCMCNAYPNSFCCNNLCDTIAAATLR + GPTCMCNAYPNSFCCNNLCDTIAAATLR	5922.360	4, 5, D	no
***P. pastors* recombinant Oo-ASP-1**			
EAEAGFCCPADLNQTDEAR + MDWDCNLEAK + NQPCK	5428.120	1, 6	no
AMIWPCTTPLPIDTSIPQNLAQWLLFQNSQENEVLTQTPWSWVTASLR + VCK	5869.917	2	no
GPTCMCNAYPNSFCCNNLCDTIAAATLR	2962.197	4, 5	no
VGCAHK + FPTGTNMVVSCAYGGEVLQDNEVVWDK	3568.652	3	no

Supplementary Table 1. Disulphide bond-linked or free thiol-carrying peptides, identified by mass spectrometry under non-reducing conditions.

Group	n	Cumulative EPG	Worm count	% L4
QuilA control	7	1630 (688 – 2413)	6500 (4000 – 10100)	50.3 (41.4 – 60.0)
Size-exclusion purified native Oo-ASP-1	7	972 (338 – 1575) *	6940 (5050 – 8800)	65.9 (51.5 – 82.7) **

Supplementary Table 2: Overview of parasitological parameters obtained after the size-exclusion native Oo-ASP-1 immunisation-infection study. n, number of animals; EPG, mean cumulative eggs per gram faeces; % L4, percentage of L4 worms observed in post-necropsy worm counting; All values represent arithmetic means (+ experimentally observed range). *P < 0.05; **P < 0.01
